# A novel peptide encoded by circ‐SLC9A6 promotes lipid dyshomeostasis through the regulation of H4K16ac‐mediated CD36 transcription in NAFLD

**DOI:** 10.1002/ctm2.1801

**Published:** 2024-08-06

**Authors:** Yue Wang, Xinyao Tian, Zhecheng Wang, Deshun Liu, Xuzi Zhao, Xin Sun, Zuoyu Tu, Zekuan Li, Yan Zhao, Shusen Zheng, Jihong Yao

**Affiliations:** ^1^ Department of Pharmacology Dalian Medical University Dalian China; ^2^ Department of Surgery Division of Hepatobiliary and Pancreatic Surgery The Second Affiliated Hospital Zhejiang University School of Medicine Hangzhou China; ^3^ Department of Surgery Division of Hepatobiliary and Pancreatic Surgery The First Affiliated Hospital Zhejiang University School of Medicine Hangzhou China; ^4^ Department of General Surgery The Second Affiliated Hospital of Dalian Medical University Dalian China; ^5^ Department of Hepatobiliary and Pancreatic Surgery Department of Liver Transplantation Shulan (Hangzhou) Hospital Hangzhou China

**Keywords:** circ‐RNA translation, h4k16ac, lipid dyshomeostasis, nuclear transport, transcription

## Abstract

**Background:**

As the leading cause of end‐stage liver disease, nonalcoholic fatty liver disease (NAFLD) is mainly induced by lipid dyshomeostasis. The translation of endogenous circular RNAs (circRNAs) is closely related to the progression of various diseases, but the involvement of circRNAs in NAFLD has not been determined.

**Methods:**

Combined high‐throughput circRNA profiles were used to identify circRNAs with translational potential. The underlying molecular mechanisms were investigated by RNA sequencing, pull‐down/MS and site‐specific mutagenesis.

**Results:**

In this study, we focused on circ‐SLC9A6, an abnormally highly expressed circRNA in human and mouse liver tissue during NAFLD development that exacerbates metabolic dyshomeostasis in hepatocytes by encoding a novel peptide called SLC9A6‐126aa in vivo and in vitro. YTHDF2‐mediated degradation of m6A‐modified circ‐SLC9A6 was found to be essential for the regulation of SLC9A6‐126aa expression. We further found that the phosphorylation of SLC9A6‐126aa by AKT was crucial for its cytoplasmic localization and the maintenance of physiological homeostasis, whereas high‐fat stress induced substantial translocation of unphosphorylated SLC9A6‐126aa to the nucleus, resulting in a vicious cycle of lipid metabolic dysfunction. Nuclear SLC9A6‐126aa promotes transcriptional activation of the target gene CD36 and enhances its occupancy of the CD36 promoter locus by regulating MOF‐mediated histone H4K16 acetylation. Hepatic CD36 depletion significantly ameliorated hyperactivated MAPK signalling and lipid disturbance in SLC9A6‐126aa transgenic mice. Clinically, increasing levels of SLC9A6‐126aa were observed during NAFLD progression and were found to be positively correlated with the CD36 and MAPK cascades.

**Conclusion:**

This study revealed the role of circ‐SLC9A6‐derived SLC9A6‐126aa in the epigenetic modification‐mediated regulation of lipid metabolism. Our findings may provide promising therapeutic targets for NAFLD and new insights into the pathological mechanisms of metabolic diseases.

**Highlights:**

- Under normal circumstances, driven by m6A modification, YTHDF2 directly recognizes and degrades circ‐SLC9A6, thereby inhibiting the translation of SLC9A6‐126aa.
- Additionally, AKT1 phosphorylates and inhibits the nuclear translocation of SLC9A6‐126aa.
- In NAFLD, lipid overload leads to YTHDF2 and AKT1 deficiency, ultimately increasing the expression and nuclear import of SLC9A6‐126aa.
- Nuclear SLC9A6‐126aa binds directly to the CD36 promoter and initiates CD36 transcription, which induces lipid dyshomeostasis.

## Graphical abstract

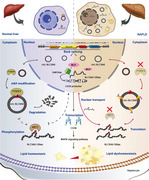

## 1 INTRODUCTION

Approximately one‐quarter of the global population suffers from NAFLD, which is a significant cause of increased liver disease‐related mortality worldwide and leads to increased susceptibility to multisystem disease and even infectious diseases.^1^, ^2^ The global prevalence of NAFLD is expected to reach 56% by 2030.^3^ However, there are currently no approved treatments for this disease,^4^ and significant efforts are thus underway to develop innovative therapies.

Hepatic lipid dyshomeostasis, an imbalance between lipid storage (fatty acid uptake and de novo adipogenesis) and lipid removal (very low‐density lipoprotein secretion and fatty acid oxidation), has emerged as the primary driver of NAFLD and causes systemic blood lipid abnormalities, oxidative stress and an excessive inflammatory response.^5^ Improving lipid dyshomeostasis has been shown to reduce the risk of NAFLD through targeted gene editing, pharmacological interventions, and gut microbiome regulation.^6^ Hence, novel molecular therapies targeting lipid metabolism are essential for a better exploration of specific upstream regulatory mechanisms.

Circular RNA (circRNA) is a single‐stranded endogenous RNA that generates a covalently closed loop through back splicing, has stable properties and exhibits high species conservation.^7^ Notably, as molecular sponges of microRNAs or conjugates of RNA‐binding proteins, circRNAs are considered promising targets for lipid metabolism‐related diseases ^8^, ^9^. Despite lacking a 5′ cap and 3′ end structures, circRNAs can be translated by recruiting ribosomes to the cytoplasm, as confirmed by several exciting studies. The coding mechanism of circRNAs with an open reading frame (ORF) depends on the internal ribosome entry site (IRES) and may be regulated by N6‐methyladenosine (m6A) modification.^10^ To date, functional peptides encoded by circRNAs (such as cGGNBP2‐184aa and Nlgn173) have been implicated in various diseases, revealing the hidden and vast potential of circRNA translation.^11^, ^12^ However, whether circRNAs can be translated into functional peptides in NAFLD is unclear.

In this study, we found that the protein‐coding potential of circ‐SLC9A6 is positively correlated with the progression of NAFLD. A novel 126‐amino‐acid protein, SLC9A6‐126aa, was translated by circ‐SLC9A6 in a m6A‐controlled manner via YTHDF2 regulation. As a result of escaping AKT1‐mediated phosphorylation, SLC9A6‐126aa translocates from the cytoplasm to the nucleus. Nuclear SLC9A6‐126aa exacerbates lipid dyshomeostasis by directly activating H4K16ac‐mediated CD36 transcription. Our results elucidate for the first time the pathogenic mechanism of circ‐SLC9A6‐encoded SLC9A6‐126aa, which could be an effective diagnostic marker and therapeutic target for NAFLD.

## 2 MATERIALS AND METHODS

### 2.1 Clinical samples

Liver specimens were collected from patients with biopsy‐proven NAFLD who underwent liver surgery or liver biopsy at Shulan Hospital. The study excluded individuals with viral infections, drug or toxin use, or excessive alcohol consumption. Paraffin sections of human liver tissue from patients with different degrees of steatosis (S0‐S3) were obtained from the Department of Pathology, Shulan Hospital. The patients gave informed consent at the time of recruitment. The Human Ethics Committee of Hangzhou Shulan Hospital approved this research protocol (Ethics approval number: KY2021025), which conformed to the ethical guidelines of the Helsinki Declaration. The clinical data and histologic characteristics of the samples are listed in Table S4. The detailed procedures are provided in the Supporting Information Materials and Methods.

## 3 RESULTS

### 3.1 High expression of circ‐SLC9A6, which has translational potential, is relevant to NAFLD

**FIGURE 1:**
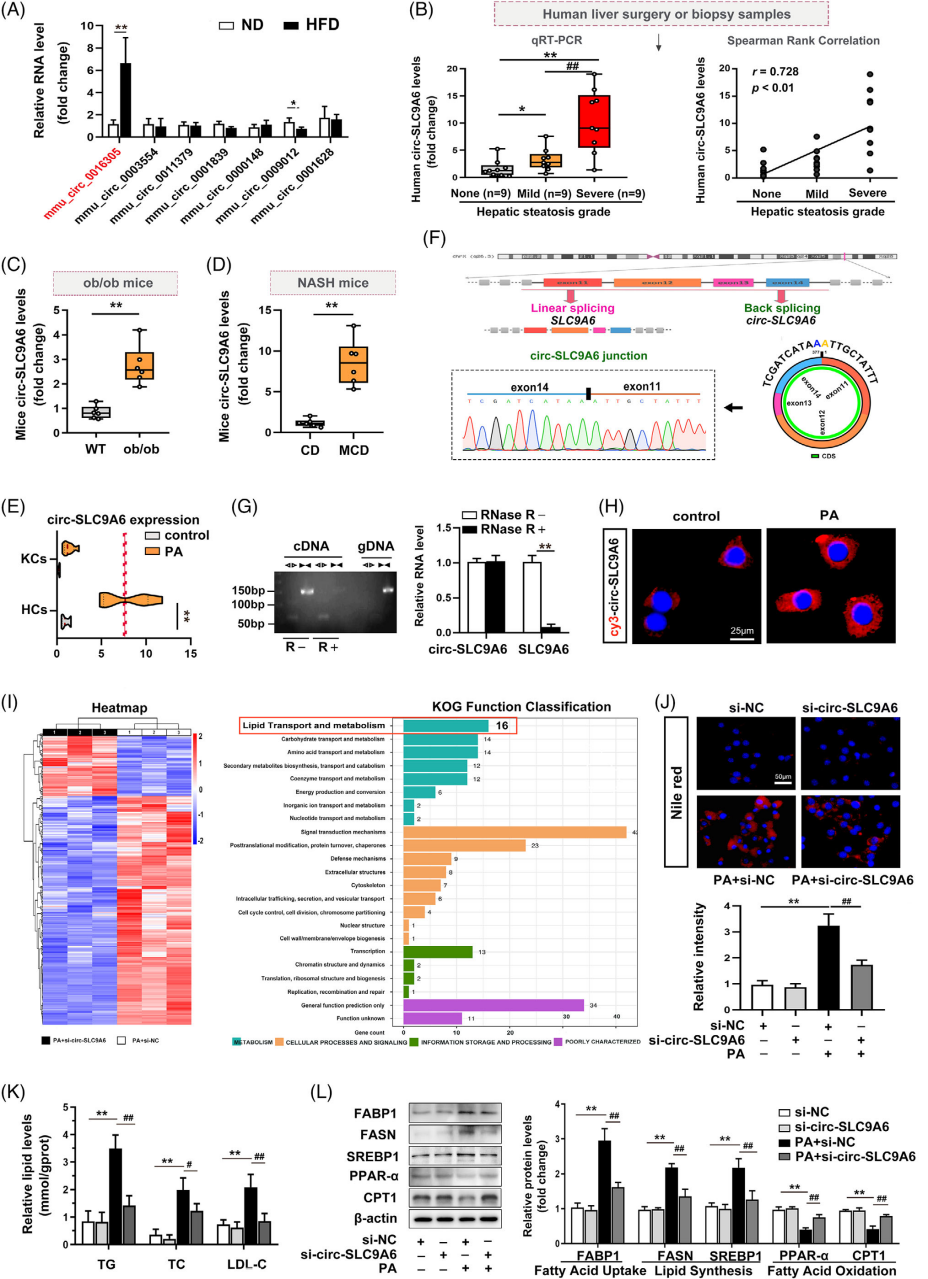
High expression of circ‐SLC9A6, which has translational potential, is relevant to NAFLD. (A) qRT‒PCR validation of the differentially expressed circRNAs; *n* = 6. (B) Circ‐SLC9A6 levels in liver tissues from patients with no (S0, *n* = 9), mild (S1, *n* = 9), or severe (S2 and S3, *n* = 9) steatosis. The correlations between the human circ‐SLC9A6 levels and the hepatic steatosis grade were analyzed by Spearman's rank correlation analysis. S0: steatosis in 0−5% of hepatocytes; S1: 6−33%; S2: 34−66%; and S3: 67−100%. (C, D) Circ‐SLC9A6 levels in liver tissues from ob/ob model mice and NASH model mice; *n* = 3. (E) Circ‐SLC9A6 levels in primary hepatocytes and Kupffer cells; *n* = 3. (F) Sketch map and Sanger sequencing. (G) RNase R degradation in AML12 cells; *n* = 3. (H) FISH of AML12 cells; *n* = 3. Scale bars = 25 µm. (I) Heatmap and EuKaryotic Orthologous Groups analysis. (J) Nile red staining of AML12 cells; *n* = 3. Scale bars = 50 µm. (K) TG, TC, and LDL‐C levels in AML12 cells; *n* = 6. (L) Protein expression in AML12 cells; *n* = 3. ^*^ *p* < 0.05, ^**^ *p* < 0.01, ^#^ *p* < 0.05, ^##^ *p* < 0.01.

To identify endogenous differentially expressed circRNAs with protein‐coding potential, we obtained high‐throughput mouse circRNA data from public RNA sequencing and microarray data (GEO: GSE94841 and PMID: 33308253^13^). The detailed screening process is shown in Figure S1A. Seven highly homologous (more than 90%) circRNAs with translational potential were selected and confirmed by qRT‐PCR analysis of NAFLD mice (Figure S1B; Figure 1A). Among these circRNAs, mmu_circ_0016305 (circ‐SLC9A6), a circRNA derived from exons 11−14 in the linear transcript of the SLC9A6 (solute carrier family 9‐member A6) gene, was significantly upregulated in NAFLD mouse livers by approximately four‐fold, in agreement with the database (Figure 1A). To determine the clinical relevance of circ‐SLC9A6, we examined the level of human circ‐SLC9A6 (hsa_circ_0139807) in liver tissue from NAFLD patients. The level of circ‐SLC9A6 was positively correlated with the progression of NAFLD in patients (Figure 1B), which was consistent with findings observed in both wild‐type and leptin‐deficient obese (ob/ob) mice and a methionine‐choline‐deficient diet‐induced steatohepatitis model (Figure 1C,D). Given the involvement of hepatocytes and Kupffer cells in the regulation of NAFLD, we isolated primary hepatocytes and Kupffer cells to further investigate the role of circ‐SLC9A6 in steatosis. Subsequent qRT‐PCR analysis revealed increased expression of circ‐SLC9A6 in primary hepatocytes under both pathological and physiological conditions (Figure 1E). Consistently, circ‐SLC9A6 expression was also upregulated in THLE2 (transformed human liver epithelial cell 2) and AML12 (alpha mouse liver 12) cells upon treatment with palmitic acid (PA) (Figure S1C,D). Hence, our research focused on investigating the functional role of circ‐SLC9A6 in hepatocytes.

Sanger sequencing and RNase R digestion confirmed the circularity of circ‐SLC9A6 (Figure 1F,G). Nucleoplasmic separation (Figure S1E) and FISH (Figure 1H) confirmed that circ‐SLC9A6 was localized mainly in the cytoplasm, providing opportunities for the recruitment of ribosomes and translation initiation factors.^12^ To elucidate the underlying molecular mechanism of circ‐SLC9A6, we analyzed the global RNA expression profile of circ‐SLC9A6‐deficient AML12 cells under PA stimulation. EuKaryotic Orthologous Groups analysis of the RNA‐seq data revealed that the function of circ‐SLC9A6 in NAFLD was centred on the lipid transport and metabolism pathway, which was the top pathway in the “metabolism” category (Figure 1I). Furthermore, Gene Ontology and Kyoto Encyclopedia of Genes and Genomes analyses revealed that circ‐SLC9A6 was involved in lipid homeostasis‐related pathways (Figure S1F,G), and these findings were validated in AML12 (Figure 1J–L) and THLE2 cells (Figure S1H). Notably, the biological function of circ‐SLC9A6 was not related to linear SLC9A6 because the specific si‐circ‐SLC9A6 did not affect the SLC9A6 mRNA levels (Figure S1I,J). These results suggest that circ‐SLC9A6, a highly stable circRNA with a closed‐loop structure, promotes NAFLD by disrupting lipid homeostasis in hepatocytes.

### 3.2 Circ‐SLC9A6 encodes a novel 126‐amino‐acid (aa) protein, SLC9A6‐126aa

**FIGURE 2:**
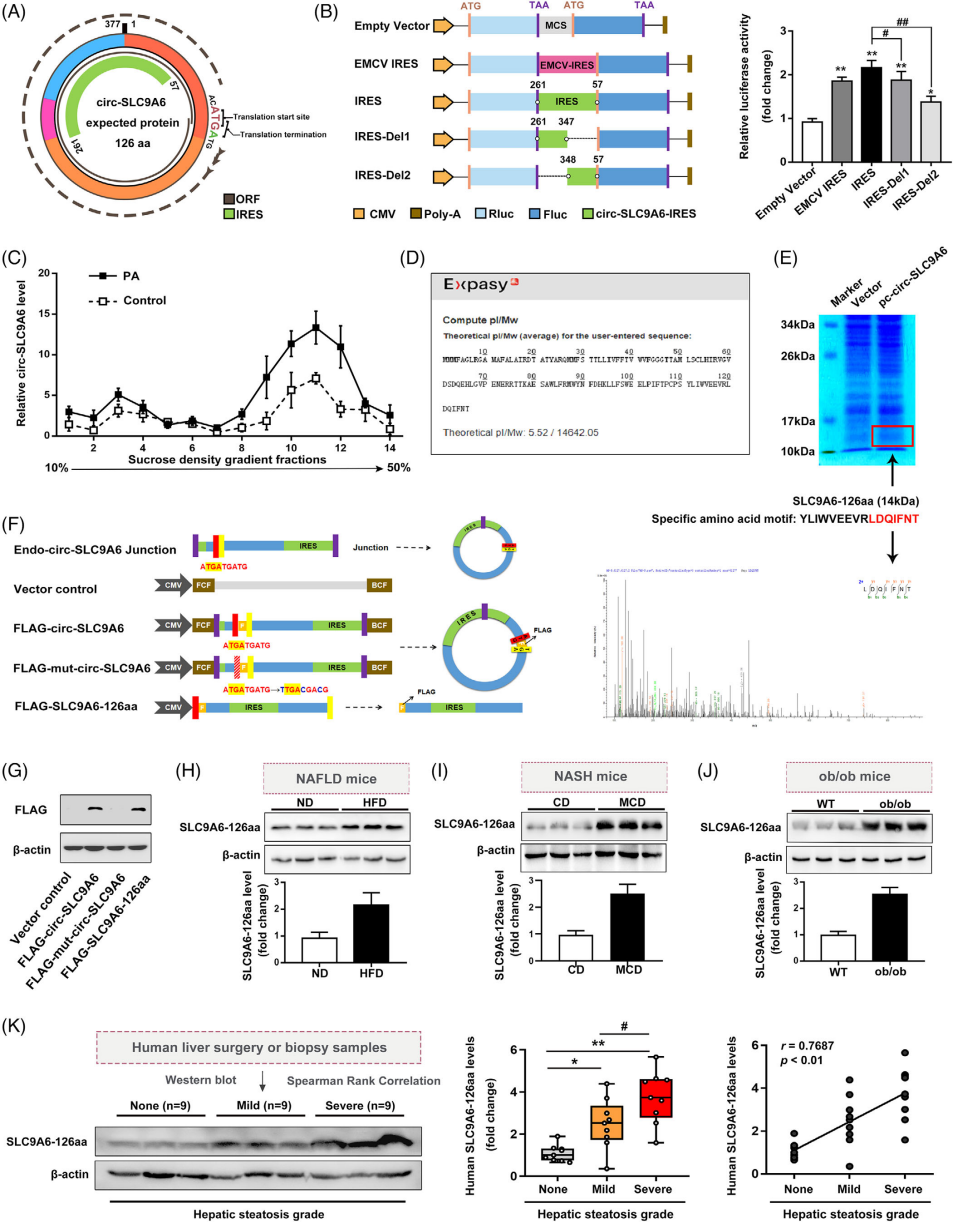
Circ‐SLC9A6 encodes a novel 126‐amino‐acid (aa) protein, SLC9A6‐126aa. (A) Schematic diagram of the translational potential of circ‐SLC9A6. (B) Schematic of five luciferase reporter vectors. The relative luciferase activity in AML12 cells is shown; *n* = 6. (C) Sucrose gradient assays; *n* = 3. (D) SLC9A6‐126aa information from the ExPASy website. (E) After AML12 cells were transfected with the vector/circ‐SLC9A6 overexpression plasmid, total proteins were separated via SDS‒PAGE. The band near 14 kDa was excised and subjected to LC‐MS/MS. (F, G) Schematic representation of the vector construction and the expression of FLAG‐SLC9A6‐126aa in AML12 cells; *n* = 3. (H–J) SLC9A6‐126aa protein levels in liver tissues from ob/ob model mice and NASH model mice; *n* = 3. (K) SLC9A6‐126aa protein levels in liver tissues from patients with no (*n* = 9), mild (*n* = 9), or severe (*n* = 9) steatosis. The correlations between the human SLC9A6‐126aa protein levels and the hepatic steatosis grade were analyzed by Spearman's rank correlation analysis. ^*^ *p* < 0.05, ^**^ *p* < 0.01, ^#^ *p* < 0.05, ^##^ *p* < 0.01.

**FIGURE 3:**
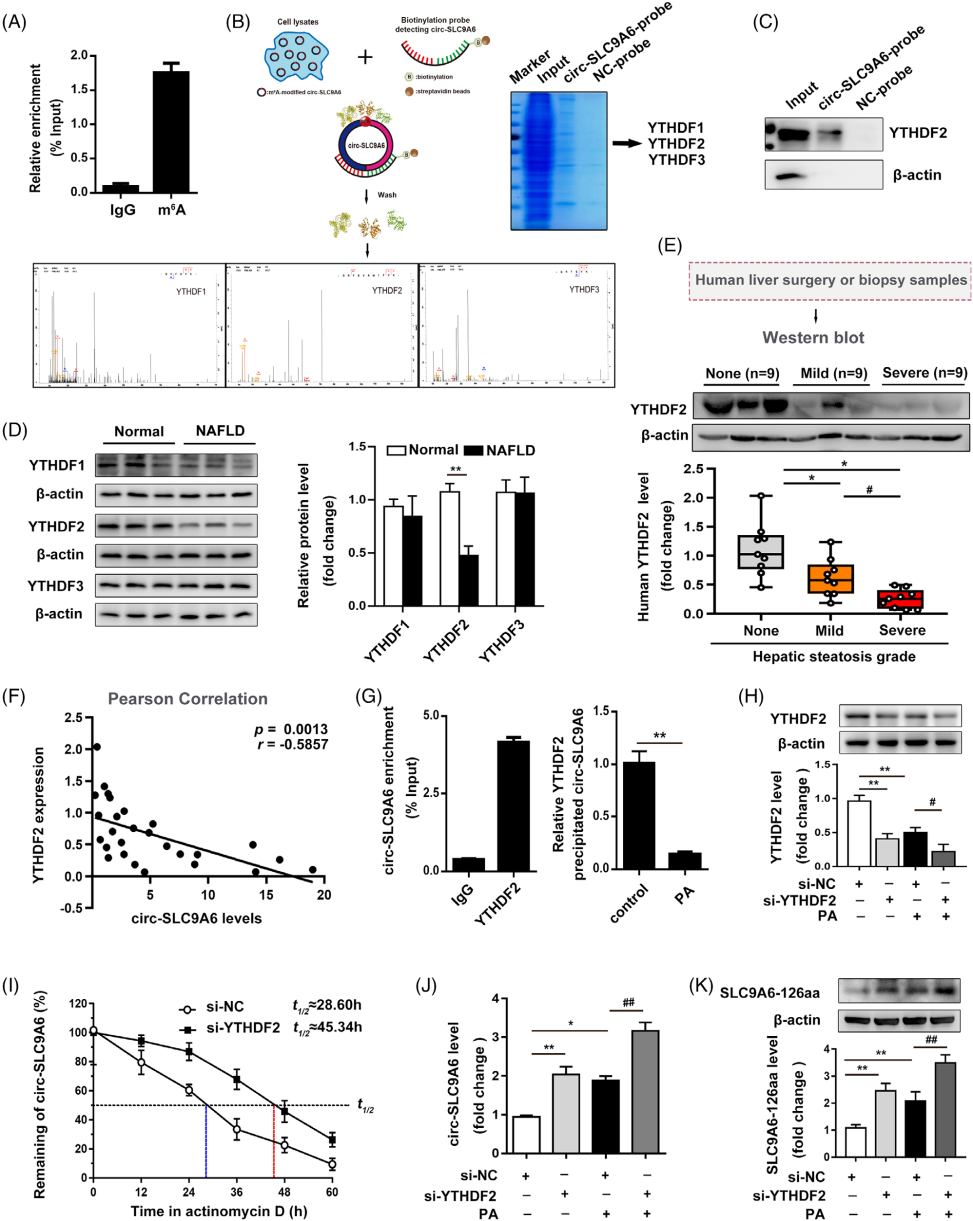
Circ‐SLC9A6 translation is regulated by YTHDF2 in a m6A‐dependent manner. (A) MeRIP assay of AML12 cells; *n* = 3. (B) Schematic representation of the circ‐SLC9A6 pull‐down procedure. Coomassie Brilliant Blue staining peptide spectrum of YTHDFs generated by LC‐MS/MS. (C) Pulldown assay with AML12 cells; *n* = 3. (D) Expression level of YTHDF1/2/3 in liver tissues of NAFLD mice; *n* = 3. (E) YTHDF2 protein levels in liver tissues from patients with no (*n* = 9), mild (*n* = 9), or severe (*n* = 9) steatosis. (F) Pearson correlation analysis between circ‐SLC9A6 and YTHDF2 expression in NAFLD patients; *n* = 27. (G) RIP assay of AML12 cells; *n* = 3. (H) YTHDF2 protein level; *n* = 3. (I) Half‐life assay of circ‐SLC9A6; *n* = 3. (J, K) Levels of circ‐SLC9A6 and SLC9A6‐126aa in AML12 cells; *n* = 3. ^*^ *p* < 0.05, ^**^ *p* < 0.01, ^#^ *p* < 0.05, ^##^ *p* < 0.01.

Moreover, novel proteins synthesized by translatable circ‐RNAs play specific roles in the onset and development of human diseases.^14^ We first analyzed the coding potential of circ‐SLC9A6 using CircPrimer software. The circ‐SLC9A6 sequence contains a hypothetical ORF and IRES (261−57 nt), which can be initiated by three tandem “ATG” codons and terminated by a “TGA” codon and encodes an undefined polypeptide of 126 aa (designated SLC9A6‐126aa) (Figure 2A). The wild‐type IRES‐transfected cells exhibited greater luciferase activity than the empty vector‐transfected cells (Figure 2B). Sucrose gradient fractionation confirmed that the PA challenge resulted in increased assembly and translation of endogenous circ‐SLC9A6 (Figure 2C). The ExPASy website predicted that SLC9A6‐126aa, which is approximately 14 kDa in length, is a highly conserved (97.62%) peptide (Figure 2D; Figure S2A). As predicted, LC‐MS/MS successfully confirmed the presence of SLC9A6‐126aa (Figure 2E). To verify that SLC9A6‐126aa was exclusively derived from circ‐SLC9A6, a FLAG‐labeled plasmid with or without translational function was constructed and transfected into AML12 cells (Figure 2F). Labelled SLC9A6‐126aa was detected at 14 kDa in the FLAG‐circ‐SLC9A6 group and the FLAG‐SLC9A6‐126aa group. However, a start codon mutation (in which the first three ATGs were mutated to TTG, ACG, and ACG) resulted in loss of the translational function of circ‐SLC9A6, and the gene was thus unable to encode SLC9A6‐126aa (Figure 2G). Although circRNAs primarily function as microRNA sponges, we found no evidence of direct binding of circ‐SLC9A6 and AOG2 (a key protein in mediating the interaction between circRNAs and miRNAs^15^) via pull‐down/MS analysis (Figure 3B). In addition, pull‐down and RNA immunoprecipitation experiments revealed that circ‐SLC9A6 exhibited a weak affinity for AGO2 (Figure S2D,E), indicating that circ‐SLC9A6 might perform its functions primarily through encoding novel protein rather than acting as a competitive endogenous RNA (ceRNA). In clinical samples, SLC9A6‐126aa protein levels were positively correlated with the progression of NAFLD, and this finding was also observed in various mouse and cell models (Figure 2H–K; Figure S2B,C), suggesting that circ‐SLC9A6 could encode SLC9A6‐126aa, which may be correlated with the exacerbation of NAFLD.

### 3.3 Circ‐SLC9A6 translation is regulated by YTHDF2 in a m6A‐dependent manner

m6A modification was recently reported to regulate circRNA translation.^16^ To investigate whether m6A modification is involved in the formation of SLC9A6‐126aa, we predicted two highly homologous m6A sites in circ‐SLC9A6 using the BERMP website (Figure S3A). MeRIP/qRT‒PCR detection confirmed that circ‐SLC9A6 was methylated in AML12 cells (Figure 3A). Interestingly, pull‐down/MS analysis revealed that the YTH domain family (including YTHDF1‐3) physically bound to circ‐SLC9A6, as confirmed by pull‐down experiments with AML12 cells (Figure 3B,C; Figure S3B,C). Notably, only YTHDF2, but not YTHDF1 or YTHDF3, was significantly downregulated in response to high‐fat diet stimulation in vivo and in vitro (Figure 3D; Figure S3D). Similarly, in clinical samples, we found that deterioration of NAFLD grading and an increase in circ‐SLC9A6 were associated with a decrease in YTHDF2 (Figure 3E,F), suggesting that YTHDF2 may be a potential m6A protein that affects the expression of circ‐SLC9A6 in NAFLD. YTHDF2 selectively recognizes specific mRNAs and regulates their degradation via the endoribonucleolytic cleavage pathway.^17^ We consistently found that PA stimulation significantly reduced the enrichment of YTHDF2 on circ‐SLC9A6 (Figure 3G). Importantly, specific silencing of YTHDF2 caused a significant increase in the SLC9A6‐126aa protein in the presence or absence of PA by inhibiting the degradation of circ‐SLC9A6 (Figure 3H–K). Therefore, the deficiency of YTHDF2 in NAFLD is responsible for enhancing the stability of circ‐SLC9A6, and this enhancement is accompanied by an increase in the translation of SLC9A6‐126aa.

### 3.4 SLC9A6‐126aa, rather than circ‐SLC9A6, aggravates lipid dyshomeostasis in vitro and in vivo

**FIGURE 4:**
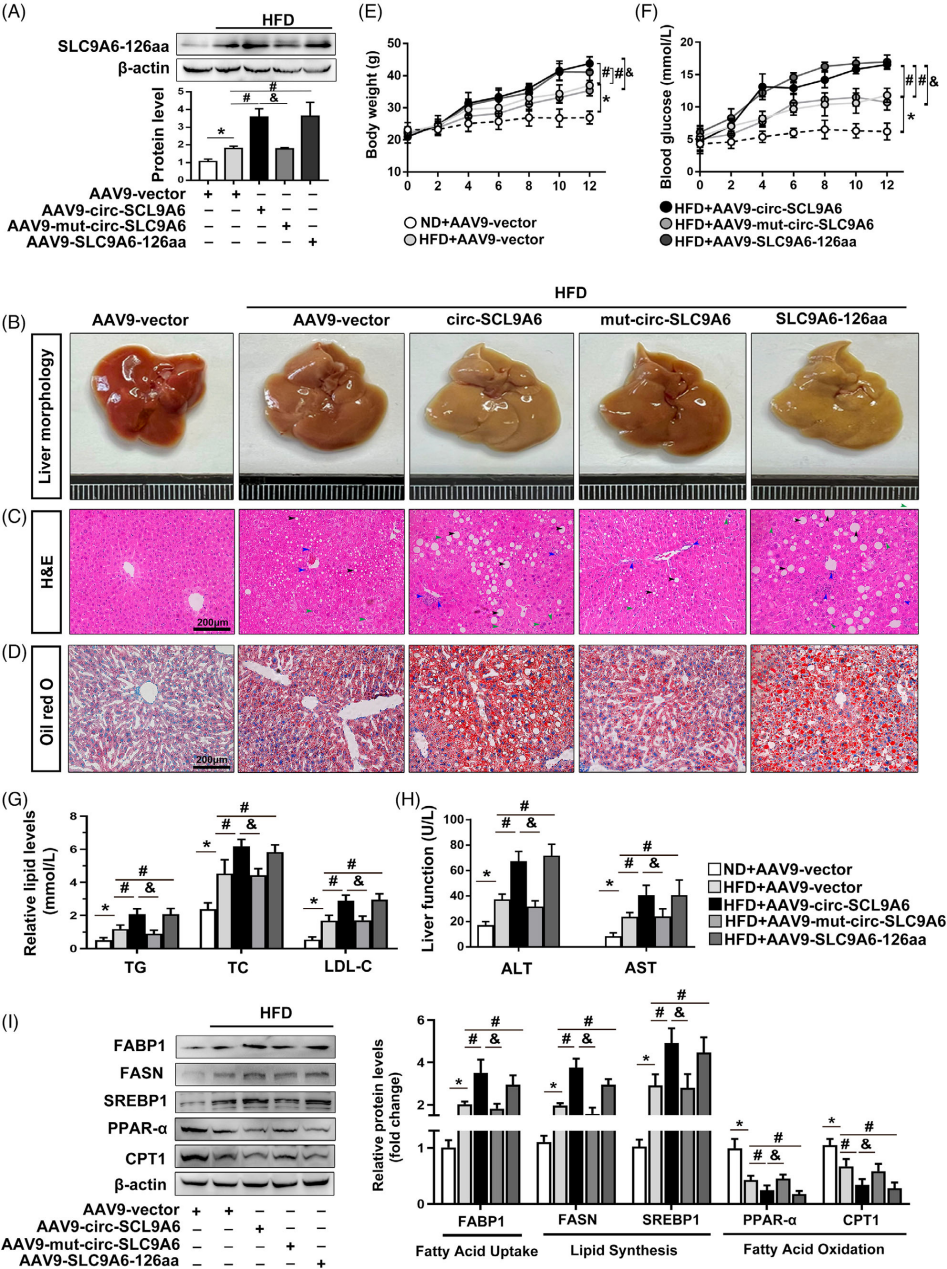
SLC9A6‐126aa, rather than circ‐SLC9A6, aggravates lipid dyshomeostasis in vitro and in vivo. (A) SLC9A6‐126aa protein level; *n* = 3. (B–D) Liver morphology, H&E staining, and Oil Red O staining. Scale bars = 200 µm. Black arrow: steatosis; green arrow: hepatocyte ballooning; blue arrow: lobular inflammatory infiltration. (E) Body weights; *n* = 6. (F) Serum glucose levels; *n* = 6. (G, H) Serum TG, TC, LDL‐C, ALT, and AST levels; *n* = 6. (I) Protein expression in mice; *n* = 3. ^*^ *p* < 0.05, ^#^ *p* < 0.05, ^&^ *p* < 0.05.

Next, we investigated whether the lipid metabolism disorders caused by circ‐SLC9A6 were dependent on SLC9A6‐126aa. Overexpression of the SLC9A6‐126aa protein was successfully detected in the liver of mice injected with AAV9‐TBG‐circ‐SLC9A6 or AAV9‐TBG‐SLC9A6‐126aa. However, a lack of translational function prevented AAV9‐TBG‐mut‐circ‐SLC9A6 from exogenously expressing SLC9A6‐126aa (Figure 4A). Hepatic histologic examination revealed that circ‐SLC9A6 or SLC9A6‐126aa, but not mut‐circ‐SLC9A6, accelerated hepatic steatosis (Figure 4B–D). Similarly, exogenous SLC9A6‐126aa markedly exacerbated HFD‐induced obesity, elevated the fasting plasma glucose levels, and resulted in abnormal serum biochemical indices similar to those observed with exogenous circ‐SLC9A6, whereas no significant change was observed in the AAV9‐TBG‐mut‐circ‐SLC9A6 group (Figure 4E–H). Compared with those in the HFD+vector group, mice injected with AAV9‐TBG‐circ‐SLC9A6 or AAV9‐TBG‐SLC9A6‐126aa exhibited more severe dyslipidemia; on the other hand, the levels of proteins associated with lipid homeostasis were unaltered in the mice injected with AAV9‐TBG‐mut‐circ‐SLC9A6 (Figure 4I). Consistent with the in vivo results, circ‐SLC9A6 or SLC9A6‐126aa significantly augmented PA‐induced lipid disorders after transfection into AML12 cells, whereas PA+mut‐circ‐SLC9A6 exhibited almost no difference in comparison with the PA+vector group (Figure S4A–D). These data suggest that SLC9A6‐126aa, which is encoded by circ‐SLC9A6, rather than the circular RNA form of circ‐SLC9A6, plays a critical role in regulating lipid metabolism in NAFLD.

### 3.5 Lipid overload triggers dynamic nuclear import of SLC9A6‐126aa

**FIGURE 5:**
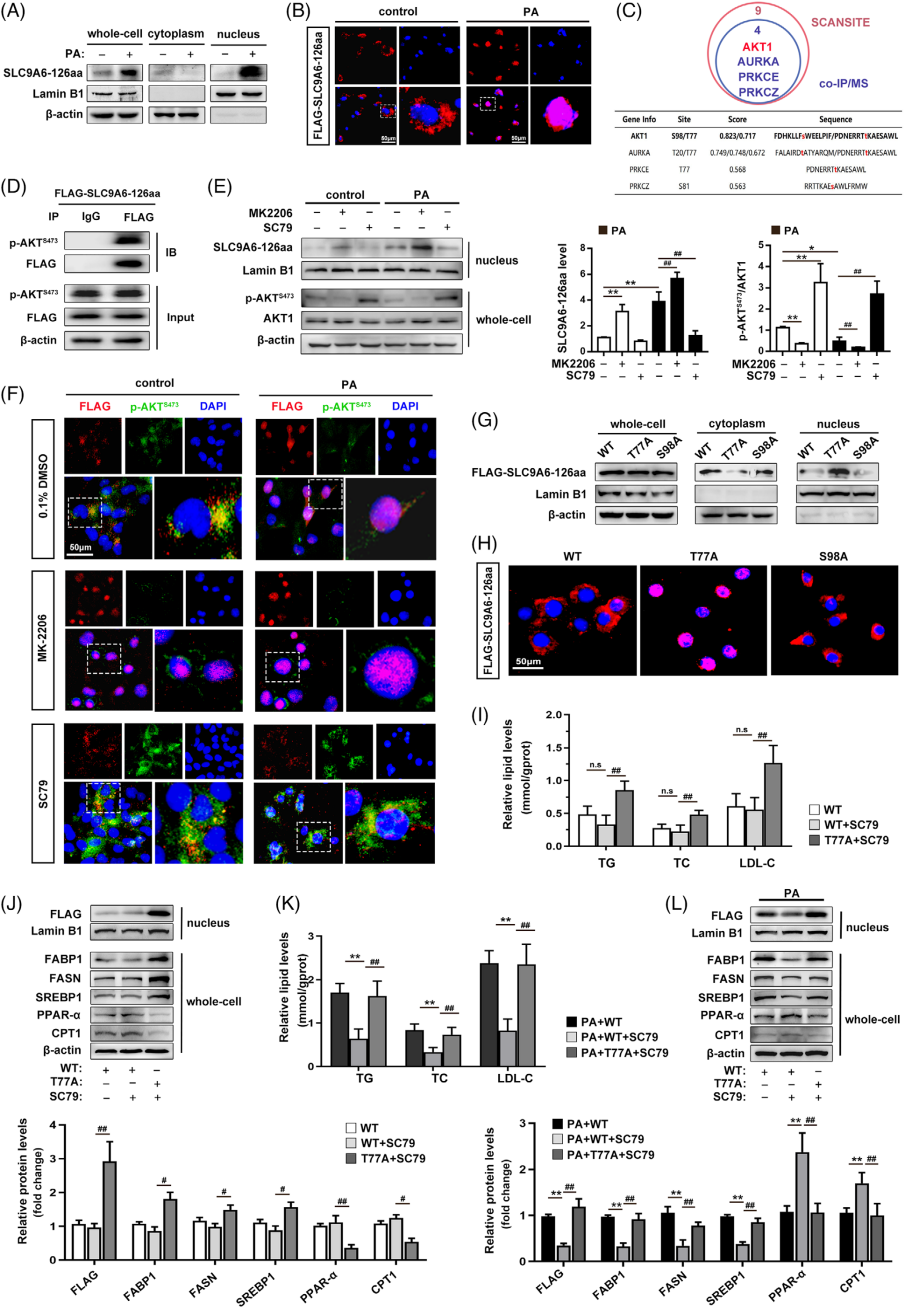
Lipid overload triggers dynamic nuclear import of SLC9A6‐126aa. (A, B) Subcellular fraction immunoblots and immunofluorescence microscopy; *n* = 3. Scale bars = 200 µm. (C) Screening of potential phosphokinases using ScanSite and co‐IP/MS. (D) Co‐IP assay with AML12 cells; *n* = 3. (E, F) AML12 cells were pretreated with MK2206 (10 µM) for 12 h or SC79 (4 µg/mL) for .5 h prior to PA‐induced stress. Subcellular fractionation immunoblots and immunofluorescence microscopy; *n* = 3. Scale bars = 200 µm. (G, H) Subcellular fractionation immunoblots and immunofluorescence microscopy; *n* = 3. Scale bars = 200 µm. (I–L) AML12 cells overexpressing WT‐ or T77A‐SLC9A6‐126aa were pretreated with SC79 (4 µg/mL) for 0.5 h in the presence or absence of PA stimulation. The TG, TC, and LDL‐C levels (*n* = 6) and related protein expression levels (*n* = 3) are shown. ^*^ *p* < 0.05, ^**^ *p* < 0.01, ^#^ *p* < 0.05, ^##^ *p* < 0.01.

To further explore the specific mechanism of SLC9A6‐126aa in lipid homeostasis, we compared the protein sequences of SLC9A6‐126aa with those of the parental gene SLC9A6 using bioinformatics websites. The absence of the b_cpa1 superfamily domain (which is responsible for the cytoplasmic pH regulation of SLC9A6 on the membrane^18^) in SLC9A6‐126aa implies that this protein may exhibit differences in localization and function compared with SLC9A6 (Figure S5A). Under normal conditions, low levels of endogenous SLC9A6‐126aa exhibited a predominantly extranuclear localization pattern. However, increased endogenous SLC9A6‐126aa was detected in the nucleus after PA treatment by subcellular fractionation (Figure 5A). Notably, FLAG‐SLC9A6‐126aa only entered the nucleus under specific pathological conditions, suggesting that high exogenous expression alone could not trigger nuclear translocation (Figure 5B). Consistent with our speculation, the above‐described results suggest that the biological effects of SLC9A6‐126aa may occur within the nucleus. Next, we identified the stress‐responsive switches that intersect with the nuclear transport of SLC9A6‐126aa. Phosphorylation is the major posttranslational modification that regulates nucleocytoplasmic trafficking under cellular stress.^19^ Based on the specific substrate motifs predicted by ScanSite, 10 phosphorylases in the cytoplasm are responsible for the nucleoplasmic translocation of SLC9A6‐126aa (Table S1). Co‐IP/MS further revealed that AKT1, AURKA, PRKCE, and PRKC2 interact with SLC9A6‐126aa. Among them, AKT1 had the most robust binding preference for SLC9A6‐126aa (Figure 5C,D). Based on its involvement in the nuclear transport of various proteins,^20^, ^21^, ^22^ AKT1 might regulate the cytoplasmic nuclear transport of SLC9A6‐126aa. Indeed, we observed that AKT1 inhibition mediated by MK2206 enhanced the nuclear translocation of SLC9A6‐126aa, whereas AKT1 activation induced by SC79 significantly increased the level of cytoplasmic SLC9A6‐126aa (Figure 5E,F). These data suggest that AKT1 is a pivotal upstream regulator of SLC9A6‐126aa nuclear trafficking in NAFLD.

There is evidence showing that the phosphorylation of certain residues affects the nuclear localization of some import proteins.^23^ Based on the consensus motifs defined for AKT1,^24^ SLC9A6‐126a was predicted to contain two putative and conserved AKT1 consensus sequences, threonine 77 (T77) and serine 98 (S98) (Figure S5B). SLC9A6‐126aa mutants of T77 or S98 (T‐to‐A, T77A or S‐to‐A, S98A) mimicking the dephosphorylated states were transfected into AML12 cells to determine the functional site. Notably, in the in vitro transport assay, SLC9A6‐126aa with the T77A mutation was spontaneously imported into the nucleus compared with the wild type, whereas SLC9A6‐126aa with the S98A mutation retained its cytoplasmic localization (Figure 5G,H). These results suggest that T77 phosphorylation impairs SLC9A6‐126aa nuclear translocation. To investigate the effect of AKT1‐mediated SLC9A6‐126aa phosphorylation in NAFLD, the AKT1 activator SC79 was incubated with AML12 cells transfected with the WT or T77A‐SLC9A6‐126aa plasmid. Apparently, activated AKT1 forced the predominant cytoplasmic localization of SLC9A6‐126aa, thereby reversing PA‐induced lipid destabilization. However, the protective effect of SC79 was attenuated following the T77 mutation (Figure 5I–L). Accordingly, AKT1 maintains cytoplasmic SLC9A6‐126aa in a T77‐dependent manner, whereas AKT1 deficiency in NAFLD exacerbates lipid dysregulation by increasing the nuclear import of SLC9A6‐126aa.

### 3.6 SLC9A6‐126aa binds to and activates CD36 promoters

**FIGURE 6:**
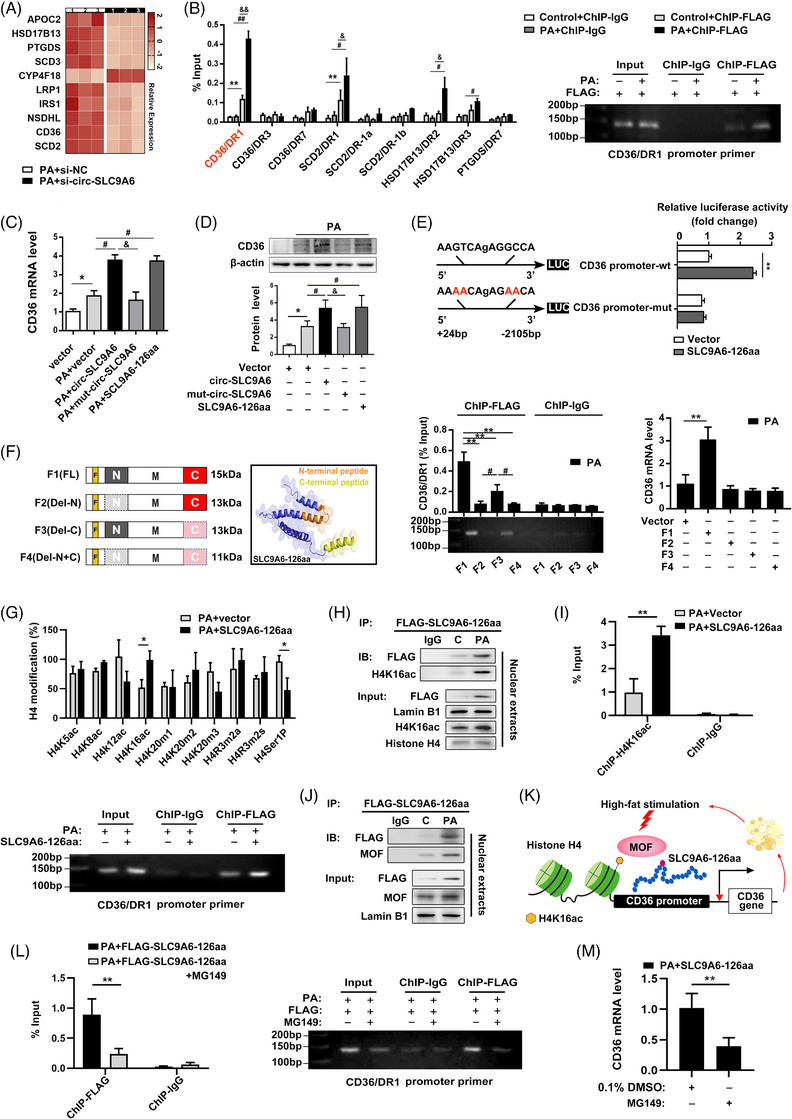
SLC9A6‐126aa binds to and activates CD36 promoters. (A) Heatmap of the top 10 lipid‐associated genes from the RNA‐seq assay. (B) ChIP‒qPCR and agarose gel electrophoresis; *n* = 3. (C, D) CD36 mRNA and protein levels; *n* = 3. (E) Luciferase reporter assays and schematic representation of the mutation sites; *n* = 6. (F) Schematic representation of the SLC9A6‐126aa domain deletions. ChIP‒qPCR and agarose gel electrophoresis (*n* = 3) and CD36 mRNA level (*n* = 3) were conducted. (G) Detection of histone H4 modifications regulated by SLC9A6‐126aa; *n* = 3. (H, J) Co‐IP assay with AML12 cells; *n* = 3. (I, L) ChIP‒qPCR and agarose gel electrophoresis results; *n* = 3. (K) Schematic representation of the SLC9A6‐126aa‐MOF‐H4K16ac tertiary complex. (M) CD36 mRNA; *n* = 3. ^*^ *p* < 0.05, ^**^ *p* < 0.01, ^#^ *p* < 0.05, ^##^ *p* < 0.01.

Multiple factors translocate to the nucleus upon cellular stress to regulate gene expression and influence epigenetic activity.^25^ Our RNA‐seq analysis showed that circ‐SLC9A6 knockdown caused broad changes in gene expression. We hypothesized that the nuclear SLC9A6‐126aa encoded by circ‐SLC9A6 may play a role in NAFLD by altering gene transcription. To elucidate the role of nuclear SLC9A6‐126aa, the top 10 lipid homeostasis‐related differentially expressed genes identified using RNA‐seq data are shown in Table S2 and were validated by qRT‒PCR (Figure 6A; Figure S6A). Among them, four genes, HSD17B13, PTGDS, CD36, and SCD2, were significantly upregulated upon SLC9A6‐126aa overexpression with PA exposure (Figure S6B). Based on sequence alignment analysis, we found that multiple palindromic consensus repeat (AGGTCA) motifs were present in the promoter regions of HSD17B13, PTGDS, CD36, and SCD2 (Figure S6C; Table S3). To investigate the possibility of SLC9A6‐126aa binding to the AGGTCA‐like sequence, we performed chromatin immunoprecipitation followed by quantitative PCR (ChIP‒qPCR). SLC9A6‐126aa bound to the promoter regions of CD36, SCD2, and HSD17B13 to varying degrees. Notably, treatment with PA resulted in FLAG‐SLC9A6‐126aa recruiting the most abundant CD36/DR1 substrates (Figure 6B). In addition, CD36 transcription was unaffected by mut‐circ‐SLC9A6 overexpression (Figure 6C,D). To further determine whether CD36/DR1 is involved in the transactivation of SLC9A6‐126aa, a luciferase reporter gene assay was performed with AML12 cells. As shown in Figure 6E, overexpression of SLC9A6‐126aa significantly enhanced the promoter activity of the wild‐type CD36 gene, whereas mutation of the binding site in the CD36 promoter abolished the effects of SLC9A6‐126aa. These results support a direct regulatory function of SLC9A6‐126aa in the induction of CD36 transcriptional activity through specific binding to the DR1 response element.

Furthermore, we intended to validate the functional region of SLC9A6‐126aa to explore the specific transcriptional regulatory mechanism involved. Bioinformatic analysis of the amino acid sequence revealed that SLC9A6‐126aa contains a functional N‐terminus and C‐terminus: the N‐terminal helix–rotation–helix (HTH) DNA‐binding motif may bind to the CD36 promoter,^12^ whereas a unique C‐terminus derived from reverse splicing may be responsible for activating CD36 transcription^26^, ^27^ (Figure S7A,B). For an in‐depth analysis of the N‐ and C‐termini of SLC9A6‐126aa, we generated various SLC9A6‐126aa deletion mutants lacking either one or two of the putative domains and stably integrated them into the genome of AML12 cells (Figure 6F). Astonishingly, the Del‐N and Del‐N+C variants completely lost the ability to bind and regulate the CD36 promoter, whereas the Del‐C variant could capture a small amount of the CD36/DR1 fragment but failed to activate CD36 transcription (Figure 6F). The difference in truncation mutations suggests that the N‐terminal and C‐terminal domains are not independent and that both are critical for the activity of SLC9A6‐126aa. Thus, these results point to the requirement of inseparable N‐ and C‐termini for SLC9A6‐126aa‐mediated CD36 transactivation.

### 3.7 SLC9A6‐126aa promotes H4K16ac deposition on the CD36 promoter by recruiting MOF

Posttranslational modifications of histones (histone PTMs) play a crucial role in DNA‐related processes, influencing the recruitment of transcriptional regulators to the active promoter region and directly impacting transcriptional efficiency.^28^ To investigate the involvement of histone PTMs in the transcriptional regulation of CD36 by SLC9A6‐126aa, we infected AML12 cells with a pcDNA3.1 vector overexpressing FLAG‐SLC9A6‐126aa for subsequent co‐IP/MS analysis. Three potential core histones, H4, H2A, and H2B, were identified via interactome analysis, and histone H4 exhibited a pronounced binding affinity (Figure S8A). Next, the impact of SLC9A6‐126aa on histone H4 modifications was evaluated by array analysis using the EpiQuick Histone H4 modification multiplex assay. Changes in the levels of two histone modifications (H4K16ac and H4Ser1P) were detected upon overexpression of SLC9A6‐126aa (Figure 6G). Considering the role of H4K16ac modification in promoting promoter activation by regulating nucleosome accessibility and its involvement in the epigenetic regulation of metabolic disorders,^29^ we hypothesized that H4K16ac modification might direct SLC9A6‐126aa to regulate CD36 transcription. The change in H4K16ac modification was verified by Western blot analysis, which demonstrated that SLC9A6‐126aa overexpression significantly enhanced the PA‐induced H4K16ac levels (Figure S8B). Co‐IP experiments confirmed the higher levels of direct interaction between SLC9A6‐126aa and H4K16ac in lipid‐overloaded hepatocytes (Figure 6H; Figure S8C). We then sought to determine whether SLC9A6‐126aa could facilitate the deposition of H4K16ac on the CD36 promoter. ChIP‒qPCR analysis showed that overexpression of SLC9A6‐126aa significantly enhanced the enrichment of H4K16ac in the CD36/DR1 response element (Figure 6I), suggesting that CD36 transcriptional activation may be mediated by SLC9A6‐126aa‐mediated H4K16ac deposition.

To elucidate the molecular mechanism underlying the regulation of the H4K16ac levels by SLC9A6‐126aa, we aimed to identify other interacting regulators by reciprocal co‐IP assays. Intriguingly, MOF, an acetyltransferase known for its role in regulating H4K16 acetylation,^30^ was identified in SLC9A6‐126aa‐immunoprecipitated nuclear protein complexes (Figure 6J; Figure S8D). We further found that fatty acid stimulation led to an increase in the binding affinity between SLC9A6‐126aa and MOF, indicating enhanced formation of the SLC9A6‐126aa‐MOF‐H4K16ac tertiary complex in the nucleus under conditions of lipid overload (Figure 6J,K). Notably, the treatment of AML12 cells with MG149, a potent inhibitor that targets MOF and suppresses H4K16ac, resulted in the inhibition of the binding of SLC9A6‐126aa to the CD36 promoter and thereby in the downregulation of CD36 expression (Figure 6L,M). These findings validate our hypothesis that SLC9A6‐126aa regulates CD36 expression by facilitating MOF‐mediated H4K16ac deposition on the CD36 promoter.

### 3.8 CD36 deficiency reverses SLC9A6‐126aa overexpression‐exacerbated lipid dyshomeostasis in NAFLD

**FIGURE 7:**
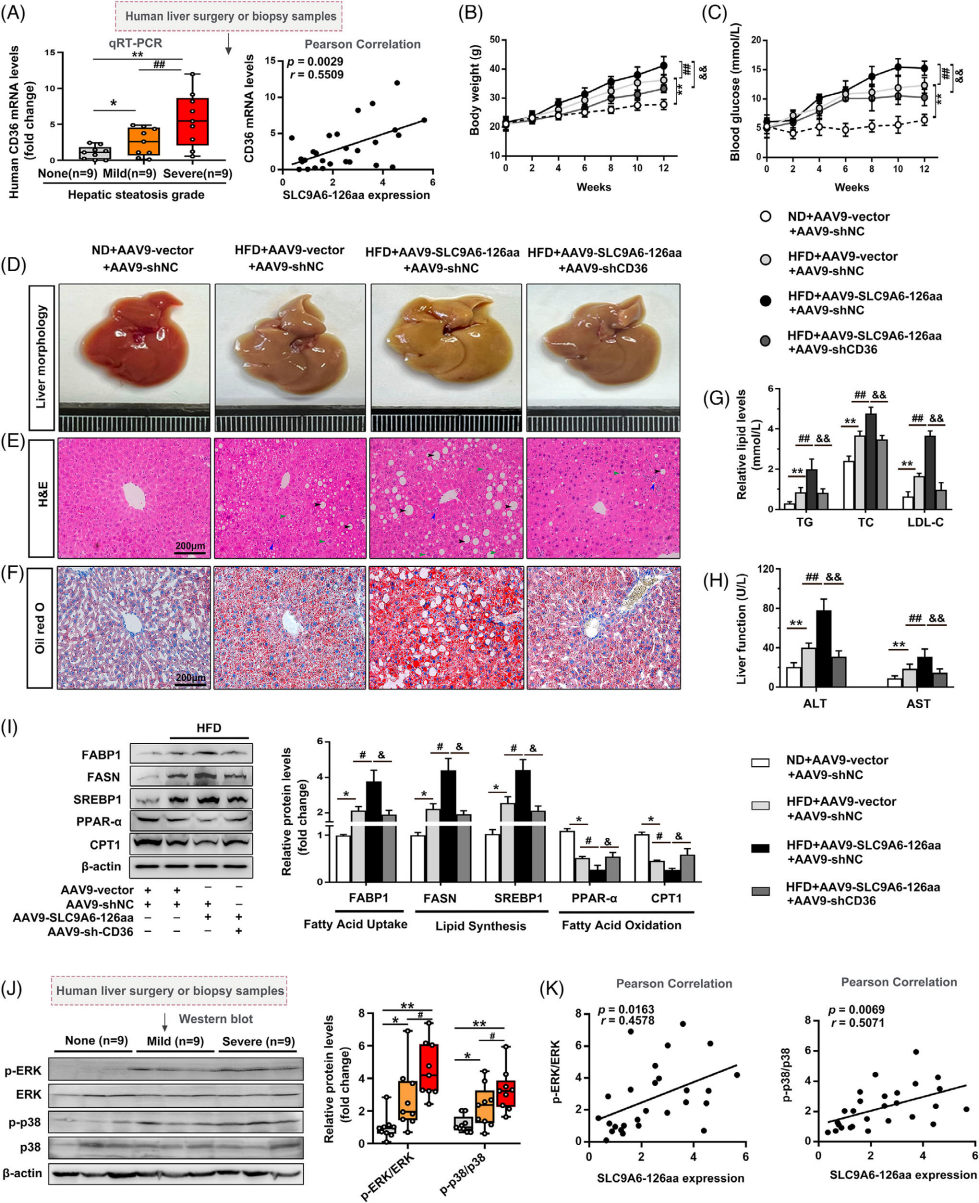
CD36 deficiency reverses SLC9A6‐126aa overexpression ‐exacerbated lipid dyshomeostasis in NAFLD. (A) CD36 mRNA levels in liver tissues from patients with no (*n* = 9), mild (*n* = 9), or severe (*n* = 9) steatosis. Pearson correlation analysis between SLC9A6‐126aa expression and CD36 mRNA expression in NAFLD patients was conducted; *n* = 27. (B) Body weights; *n* = 6. (C) Serum glucose levels; *n* = 6. (D–F) Liver morphology, H&E staining, and Oil Red O staining; scale bars = 200 µm. Black arrow: steatosis; green arrow: hepatocyte ballooning; blue arrow: lobular inflammatory infiltration. (G, H) Serum LDL‐C, TG, TC, ALT, and AST levels; *n* = 6. (I) Protein expression in mice; *n* = 3. (J) p‐p38 and p‐ERK levels in liver tissues from patients with no (*n* = 9), mild (*n* = 9), or severe (*n* = 9) steatosis. ^*^ *p* < 0.05, ^**^ *p* < 0.01, ^##^ *p* < 0.01, ^&^ *p* < 0.05, ^&&^ *p* < 0.01.

Clinical studies have confirmed the pathological significance of CD36 in NAFLD patients based on its high expression in the liver.^31^ Figure 7A shows that CD36 mRNA expression was increased in the livers of NAFLD patients and was correlated with high SLC9A6‐126aa expression, suggesting that SLC9A6‐126aa may mediate CD36 transcription and contribute to NAFLD progression. Our results further confirmed that the increases in the body weight and blood glucose levels in AAV9‐TBG‐SLC9A6‐126aa mice were abolished by liver‐specific knockdown of CD36 (Figure 7B,C). Morphological examination and histological staining revealed that CD36 deficiency abrogated the increases in hepatic steatosis, inflammation and liver injury induced by SLC9A6‐126aa overexpression (Figure 7D–F). In addition, CD36 knockdown antagonized the SLC9A6‐126aa‐induced increase in the serum levels of biochemical indices and lipid metabolism disturbance in HFD‐fed mice (Figure 7G–I). Similar results were observed in vitro (Figure S9A–C). Thus, these data suggest that CD36 is an indispensable downstream target of SLC9A6‐126aa in NAFLD. Previous studies have confirmed that CD36 expression is correlated with MAPK signalling activation.^32^ Our gene set enrichment analysis showed enrichment of MAPK signalling in SLC9A6‐126aa‐deficient AML12 cells (Figure S10A,B), further supporting the involvement of SLC9A6‐126aa in the regulation of lipid homeostasis through the CD36‐MAPK cascades. Figure 7J,K showed that the activation of the p38‐MAPK and ERK‐MAPK signalling pathways was sustained in human samples during the progression of steatosis and exhibited a significant positive correlation with SLC9A6‐126aa expression. CD36 knockdown counteracted the SLC9A6‐126aa‐induced upregulation of p‐p38 and p‐ERK‐MAPK expression both in vivo and in vitro (Figure S10C,D). To further investigate whether this pathway is required for SLC9A6‐126aa‐induced lipid dyshomeostasis, specific inhibitors targeting p38 (SB‐203580) and ERK (GDC‐2035806) were used to pretreat AML12 cells prior to PA treatment. As shown in Figure S10A–C, pharmacological inhibition of either the ERK or p38 pathway, especially combined inhibition, significantly ameliorated SLC9A6‐126aa‐induced lipid metabolism disorders. In summary, the detrimental effect of excessive SLC9A6‐126aa, which is encoded by circ‐SLC9A6, may be dependent mainly on the activation of CD36 transcription and the downstream MAPK signaling pathway during the development of NAFLD, which may lead to therapeutic targets for clinical therapy.

## 4 DISCUSSION

An abnormal lipid balance is believed to be the root cause of NAFLD, resulting in hepatocyte damage, such as stress, inflammation, and apoptosis.^33^ Here, we provide the first demonstration of the translational function of circRNAs in NAFLD and uncover a novel molecular component, SLC9A6‐126aa, which is derived from circ‐SLC9A6 and drives lipid dyshomeostasis in the cytoplasmic‐nuclear communication pathway.

According to recent research, circRNAs may be viable biomarkers and therapeutic targets for NAFLD.^9^ CircRNAs have been shown to encode functional peptides involved in carcinogenesis or with antitumor action.^11^, ^12^ On the basis of a comprehensive analysis of the coding circRNA expression profiles during NAFLD progression via circRNAomics from public databases, we provide evidence that circ‐SLC9A6 is critical for lipid dyshomeostasis in hepatocytes by encoding the novel protein SLC9A6‐126aa rather than functioning as a ceRNA, which is consistent with the findings reported by Zihao Pan et al.^15^ The clinical targeting of circ‐SLC9A6 and SLC9A6‐126aa in NAFLD may provide important insights into potential interventions for NAFLD‐related diseases such as type 2 diabetes, sarcopenia, and aging.^34^, ^35^

Recent studies have shown abundant m6A motifs near translation initiation sites in endogenous translatable circRNAs, suggesting that m6A methylation is involved in the translational landscape of the human circRNA genome.^16^ Our results demonstrated that the degradation of circ‐SLC9A6 is mediated by YTHDF2, which restricts the endogenous expression of SLC9A6‐126aa. These findings highlight the importance of the m6A‐mediated translation of circ‐SLC9A6 in metabolic syndrome. Proteomic data show that YTHDF2 interacts extensively with m6A proteins,^36^ suggesting that SLC9A6‐126aa degradation may be coordinated by YTHDF2 and other proteins, which is worthy of further investigation. Overall, the generation of SLC9A6‐126aa, which can be modified by YTHDF2, is largely responsible for the detrimental effects of circ‐SLC9A6 on hepatic lipid metabolism.

In our study, elevated SLC9A6‐126aa was associated with the severity of steatosis in NAFLD patients. Furthermore, liver‐restricted SLC9A6‐126aa overexpression in mice impaired lipid homeostasis, leading to increased lipid storage and metabolic disturbance. These findings support the clinical therapeutic significance of inhibiting the expression of SLC9A6‐126aa in NAFLD. Several stress response factors, such as YAP and MSN2, translocate from the cytoplasm to the nucleus to regulate gene expression and signal transduction in dephosphorylated states.^37^, ^38^ Our data indicate that the exogenous manipulation of SLC9A6‐126aa expression had a lower effect on cytoplasmic‐nuclear trafficking under homeostatic conditions, whereas a large amount of nuclear SLC9A6‐126aa was found in PA‐treated AML12 cells under lipid‐induced stress, suggesting that SLC9A6‐126aa alone is not sufficient to induce lipid metabolism‐related changes but needs to be under a specific stimulus. These findings highlight the importance of molecular communication between subcellular structures and the nucleus under physiological or pathological conditions^25^ as well as the pathogenicity of SLC9A6‐126aa. AKT1 has been reported to regulate the nuclear trafficking of multiple downstream substrates involved in cell survival and cell growth.^24^ Our results support this hypothesis because AKT1 was identified as the most likely phosphokinase for determining the spatial localization of SLC9A6‐126aa. We observed that the inactivation of AKT1 signaling in NAFLD, as previously reported,^39^ resulted in the nuclear localization of SLC9A6‐126aa. Additionally, phosphorylation of the T77 site by AKT1 strongly promoted the cytoplasmic localization of SLC9A6‐126aa. These results are consistent with previous reports showing that the phosphorylation of the p27, p21, SKP2, and FOXO proteins by AKT1 contributes to their cytoplasmic localization in human cancers.^21^ Taken together, our findings provide a molecular basis for the spatial translocation of SLC9A6‐126aa in response to high‐fat stress and identify SLC9A6‐126aa as a novel AKT1 downstream substrate that contributes to lipid dyshomeostasis in NAFLD through escape from the phosphorylation of AKT1. Other kinases may phosphorylate SLC9A6‐126aa, but this possibility requires further investigation.

The effect of CD36 on lipid metabolism homeostasis has been well demonstrated in both mice and humans.^40^ In addition, the involvement of CD36 in various disease processes, including cholesterol efflux, tumor growth and adhesion, and platelet activation, through the regulation of downstream MAPK cascades has been documented. The ERK‐ and p38‐MAPK signaling pathways play pivotal roles in the regulation of lipid metabolism in liver diseases.^32^, ^41^ In our study, CD36 was identified as a potential high‐confidence downstream target of SLC9A6‐126aa. In the setting of lipid overload, SLC9A6‐126aa overexpression resulted in hepatic lipid storage, LDL‐C secretion, and fat oxidation‐reduction, and these effects were significantly reversed by liver‐specific CD36 knockdown. The p38‐ or ERK‐MAPK cascade may respond to different cellular processes in various diseases, which can be attributed to multiple upstream/downstream effectors (such as PKD‐1 and Fyn) that mediate distinct regulatory effects on the p38‐ and ERK‐MAPK signalling pathways.^42^, ^43^ However, several recent studies have indicated that lipid metabolic homeostasis is one of the coregulatory events of the p38‐ and ERK‐MAPK cascades.^44^ Our findings confirm that SLC9A6‐126aa, a novel upstream regulator of the MAPK cascade, promotes lipid dyshomeostasis in NAFLD via CD36‐mediated phosphorylation of p38 and ERK, supporting the above notion of p38‐ and ERK‐MAPK pathways‐mediated reprogramming of lipid metabolism. Additionally, SLC9A6‐126aa expression was positively correlated with increased CD36 mRNA and p‐p38 and p‐ERK levels in the livers of NAFLD patients. Our data highlight the novel therapeutic role of targeting the SLC9A6‐126aa/CD36/MAPK axis in human NAFLD and other metabolic syndrome disorders.

Recent studies have shown that the transcriptional activation domain promotes the activation of transcription factors by enhancing the interaction between the DNA binding domain and chromatin rather than the traditional two independent modular units.^45^ Indeed, the transcriptional activation domain of NF‐κB promotes interactions with DNA sequences to activate transcription by stimulating the binding of NF‐κB dimers.^46^ In this study, we conducted a structural characterization of SLC9A6‐126aa to understand its functional properties. Strikingly, deletion of the N‐terminal HTH motif (one of the most ubiquitous motifs in transcription factors^47^) rendered SLC9A6‐126aa incapable of binding to the CD36 promoter, suggesting that the N‐terminus may function as a DNA‐binding domain. In addition, deletion of the C‐terminus of SLC9A6‐126aa not only weakened the DNA binding affinity for CD36 but also failed to initiate CD36 transcription, indicating that the C‐terminus may be responsible for transcriptional activation and cooperation with the N‐terminus. The observation that full‐length SLC9A6‐126aa enhances transcriptional regulation is consistent with the findings of previous studies showing that the C‐terminal truncation of p53 impairs both N‐terminal DNA binding and transcription,^26^ shedding light on the importance and pathological relevance of the transcriptional regulation of SLC9A6‐126aa in response to high‐fat stress.

Epigenetic modifications have been implicated in the pathogenesis of various diseases ranging from NAFLD to hepatocellular carcinoma through the regulation of gene transcription,^48^ and H4K16ac plays a pivotal role in disrupting metabolic homeostasis.^29^ H4K16ac marks actively transcribed genes and regulates chromatin assembly and remodelling processes.^49^ In this study, we identified H4K16ac as one of the predominant modes of histone H4 modification regulated by SLC9A6‐126aa. The high lipid stimulation‐induced recruitment of MOF by SLC9A6‐126aa played a crucial role in triggering H4K16ac deposition. In turn, increased levels of H4K16ac promoted a more accessible conformation that facilitated the binding of SLC9A6‐126aa to the CD36 promoter. These processes constitute a positive feedback loop, which is consistent with the current view that H4K16ac serves as an epigenetic mediator of gene activation.^49^ Given that H4K16ac is a key player in type 2 diabetes and ageing, it will be worthwhile to further investigate whether SLC9A6‐126aa/H4K16ac affects NAFLD‐related diseases.^50^ In addition to MOF, H4K16ac levels are also regulated by deacetylases (such as HDACs) in both humans and mice,^29^ suggesting that chronic imbalance of H4K16ac mediated by SLC9A6‐126aa may be associated with dysregulated acetylase or deacetylase activity or coregulation. The above evidence suggests that the extensive epigenetic regulatory function of circ‐SLC9A6‐derived SLC9A6‐126aa in the genome may depend on its regulation of histone H4 modifications.

This study has several limitations. The clinical evidence for circ‐SLC9A6 and SLC9A6‐126aa is limited, and a large clinical trial will provide valuable human somatic data for the diagnosis and therapeutic role of circ‐SLC9A6 and SLC9A6‐126aa in NAFLD. Further analysis of our RNA‐seq data revealed that SLC9A6‐126aa may be involved in other transcription‐related regulatory processes, such as chromatin structure dynamics, DNA replication, repair, and recombination. The essential role of nuclear SLC9A6‐12aa in metabolic diseases requires further investigation.

In conclusion, we report the effect and mechanism of circ‐SLC9A6 in NAFLD. Circ‐SLC9A6 is translated into a novel peptide, SLC9A6‐126aa, which contains specific motifs and functional domains. SLC9A6‐126aa can translocate to the nucleus under lipid‐induced pressure by escaping AKT1 phosphorylation and thereby directly activating the CD36 promoter to exacerbate lipid dyshomeostasis. Furthermore, liver tissues from patients with NAFLD also accumulate SLC9A6‐126aa derived from circ‐SLC9A6, suggesting a potential pathogenic role for this process in human lipid metabolism‐related disorders.^34^ Our findings hold promise for the development of effective therapeutic interventions and diagnostic criteria for diseases associated with NAFLD.

## AUTHOR CONTRIBUTIONS

Jihong Yao, Shuzhen Zheng, Yan Zhao, and Yue Wang designed and conceived this project. Yue Wang and Xinyao Tian performed the experiments, analyzed the data, and wrote the manuscript. Zhecheng Wang, Deshun Liu, Xuzi Zhao, Xin Sun, Zuoyu Tu, and Zekuan Li analyzed and interpreted the data. Yue Wang, Yan Zhao, and Xinyao Tian collected the human samples and analyzed the data. Jihong Yao and Yan Zhao supervised the study. All the authors contributed to and approved the manuscript.

## CONFLICT OF INTEREST STATEMENT

The authors declare no conflict of interest.

## FUNDING INFORMATION

This work was funded by the National Natural Science Foundation of China (no. 82273993, 82200658).

## ETHICAL APPROVAL

All animal experiments were approved by the Ethics Committee of Dalian Medical University (ethics approval number: AEE20005) and conducted in accordance with the ARRIVE Guidelines for the Management and Use of Laboratory Animals, and all human studies were approved by the Human Ethics Committee of Hangzhou Shulan Hospital (ethics approval number: KY2021025) and conducted in accordance with the ethical guidelines of the Helsinki Declaration.

## Supporting information

Supporting Information

## Data Availability

All data needed to evaluate the conclusions in the paper are present in the paper and/or the Supporting Information Materials. The RNA‐seq data has been deposited in GEO (https://www.ncbi.nlm.nih.gov/geo/) with the accession number GSE237554. The pull‐down/MS and co‐IP/MS data were uploaded into the ProteomeXchange Consortium (http://proteomecentral.proteomexchange.org) via the iProX partner repository with the dataset identifiers PXD043698 and PXD043699.
