# Supplementary material for: A novel peptide encoded by circ‐SLC9A6 promotes lipid dyshomeostasis through the regulation of H4K16ac‐mediated CD36 transcription in NAFLD
Source: Clin Transl Med. 2024 Aug 6;14(8):e1801. doi: 10.1002/ctm2.1801 (PMC11303264; doi:10.1002/ctm2.1801)
Supplement: Supplementary file 1 — Supporting Information [file CTM2-14-e1801-s001.docx]

**A novel** **peptide encoded by circ-SLC9A6 promotes lipid dyshomeostasis through the** **regulation of H4K16ac-mediated CD36 transcription in NAFLD**

Yue Wang^1#^, Xinyao Tian^2,3#^, Zhecheng Wang^1^, Deshun Liu^4^, Xuzi Zhao^4^, Xin Sun^1^, Zuoyu Tu^1^, Zekuan Li^3^, Yan Zhao^1*^, Shusen Zheng^3,5*^, Jihong Yao^1*^

**Affiliations:**

^1^Department of Pharmacology, Dalian Medical University, Dalian, China.

^2^Division of Hepatobiliary and Pancreatic Surgery, Department of Surgery, The Second Affiliated Hospital, Zhejiang University School of Medicine, Hangzhou, China.

^3^Division of Hepatobiliary and Pancreatic Surgery, Department of Surgery, The First Affiliated Hospital, Zhejiang University School of Medicine, Hangzhou, China.

^4^Department of General Surgery, The Second Affiliated Hospital of Dalian Medical University, Dalian, China.

^5^Department of Hepatobiliary and Pancreatic Surgery, Department of Liver Transplantation, Shulan (Hangzhou) Hospital, Hangzhou, China.

^#^These authors contributed equally to this work.

**^*^Corresponding authors:**

Jihong Yao, Department of Pharmacology, Dalian Medical University, Dalian, 116044, China. E-mail: yaojihong65@hotmail.com;

Yan Zhao, Department of Pharmacology, Dalian Medical University, Dalian, 116044, China. E-mail: zhaoyan2019@dmu.edu.cn;

Shusen Zheng, The First Affiliated Hospital, Zhejiang University School of Medicine, Hangzhou, 310003, China.E-mail: [shusenzheng@zju.edu.cn](mailto:shusenzheng@zju.edu.cn).

**Table of Contents**

[Materials and methods 1](#_Toc2484)

[Supplementary figures 8](#_Toc25619)

[Table S1-S7 1](#_Toc1925)9

**Materials and methods**

**Animal experiments**

C57BL/6 mice (male, 8 weeks, 22±2 g) were obtained from the SPF Animal Laboratory Center of Dalian Medical University. In this study, a high-fat diet (60% fat energy supply, D12492) was fed to the mice for 12 weeks to establish a stable model of NAFLD. Male homozygous ob/ob mice (6 weeks) were used as a leptin-deficient obese model and wild-type littermates as control (WT) (Changsheng Biotechnology Co., Ltd.,Liaoning,China). A methionine-choline-deficient (MCD) diet was fed to the mice for 6 weeks to establish a stable model of NASH. Adeno-associated virus 9 (AAV9) was provided by Hanbio Biotechnology Co., Ltd. (Shanghai, China). Briefly, circ-SLC9A6, mut-circ-SLC9A6, SLC9A6-126aa or CD36 shRNA were driven by the liver-specific thyroxin binding globulin (TBG) promoters and packaged into AAV9 (shRNA sequences are listed in Table S5). The mice were injected with AAV9-vector, AAV9-TBG-circ-SLC9A6,AAV9-TBG-mut-circ-SLC9A6,AAV9-TBG-SLC9A6-126aa or AAV9-TBG-shCD36 via a single tail vein injection 3 weeks prior to high-fat feeding. This study strictly adhered to the ARRIVE guidelines for the management and use of laboratory animals and was approved by the Ethics Committee of Dalian Medical University (Ethics approval number: AEE20005).

**Cell culture and transfection**

AML12 cells (CTCC-001-0336, ATCC, VA, USA) were cultured in DMEM: F12 medium supplemented with 10% FBS, dexamethasone and ITTS. THLE2 cells (CTCC-004-0030, ATCC) were cultured in BEGM supplemented with 10% FBS.

FLAG-circ-SLC9A6 was chemically synthesized and cloned into the pcDNA3.1 vector containing artificial flanking sequences. FLAG tag was inserted after the start codon of the putative ORF. FLAG-circ-SLC9A6, FLAG-SLC9A6-126aa, and FLAG-mut-SLC9A6-126aa (the first three ATGs were mutated to TTG, ACG, and ACG, respectively) plasmids were constructed by GenePharma (Suzhou, China). Sequences of potential IRES or IRES with truncated mutants were amplified and inserted between Renilla luciferase (RLuc) and Firefly luciferase (FLuc) on a P-Luc2-IRES-Report vector (Geneseed Biotechnology Co., Ltd., Guangzhou, China). Wild-type or mutant CD36 promoters were synthesized and subcloned into the reporter vector GV354 (GeneChem Co., Ltd., Shanghai, China). The mouse CD36 promoter is shown in Table S6. Plasmids or siRNAs were transfected into AML12 cells or THLE2 cells using Lipofectamine 3000 (Invitrogen, Carlsbad, CA, USA). The specific siRNA sequences are listed in Table S5.

**RNA sequencing**

High-throughput sequencing and analyses of mRNAs were carried out by Wefind Biotechnology Co., Ltd. (Wuhan, China). After the samples were qualified, the library was constructed according to the Illumina sample preparation process within the RNA-seq protocol. The purified double-stranded cDNA was selected based on the fragment size using AMPure XP Beads. DESeq2 software was used to analyze the differential expression between the sample groups.

**LC‒MS/MS analysis**

Protein profiling of captured coprecipitated complexes in gels or magnetic beads was performed by Bioprofile Technology (Shanghai, China). Briefly, C18 StageTip was used for desalting the peptide for LC‒MS/MS analysis. The LC‒MS/MS data were analyzed using MaxQuant 1.6.1.0 software. Peptide-spectrum-matched and protein search results were exported based on a false discovery rate < 1%.

**Immunoprecipitation and circRNA pull-down**

IP detection was performed using protein A/G immunoprecipitation magnetic beads (Bimake, Houston, TX, USA). Briefly, cells were lysed in IP buffer (Beyotime Biotechnology, Shanghai, China) and incubated with protein A/G magnetic beads that had been conjugated to the primary antibody.

The Pierce^TM^ Magnetic RNA‒Protein Pull-Down Kit (#20164, Thermo Fisher Scientific, CA, USA) and a biotin-labeled circ-SLC9A6 probe (GenePharma) were used in the pull-down assay. The biotin-labeled circ-SLC9A6 probe sequence was CTTCCCAAGAAAATAGCAATATTTATGATCGAA GTTGTACCA.

**RNA isolation and quantitative real-time PCR**

TRIzol reagent (TaKaRa, Japan) was used for the extraction of total RNA. A reverse transcription kit (AG11711, Accurate Biotechnology Co., Ltd., Hunan, China) was used for the synthesis of cDNA templates. The RNA abundance was detected using SYBR Green. Real-time PCRs were conducted using a Bio-Rad CFX96TM Touch Real-Time PCR System (CA, USA). β-actin was used as housekeeping gene for qRT‒PCR validation. The primer sequences are listed in Table S6.

**RNase R treatment**

RNase R (RNR07250, Epicenter Biotechnologies, WI, USA) was used for the degradation of linear mRNA. Briefly, total RNA (20 μg) extracted from AML12 cells was exposed to RNase R digestion (20 U/μL).

**Sanger sequencing**

The cDNA from AML12 cells was amplified by PCR using a primer specifically targeting circ-SLC9A6. Sanger sequencing was performed by BioEngineering Co., Ltd. (Shanghai, China), using circ-SLC9A6 sequencing primers (see Table S6).

**Nuclear and cytoplasmic RNA extraction**

RNA fractions were isolated using the PARIS™ kit (AM1556, Thermo Fisher Scientific, Waltham, MA, USA). After lysis, the cells were centrifuged at 500×g. The supernatant was the cytoplasmic fraction. The pellets were then collected with Nuclear Fraction Buffer.

**Subcellular protein fractionation**

Nuclear and cytoplasmic proteins were extracted using the Cell Fractionation Kit (TransGen Biotech, Beijing, China).

**Fluorescence in situ hybridization (FISH)**

A fluorescence in situ hybridization kit (GenePharma, Suzhou, China) was used for FISH. The anti-circ-SLC9A6 oligodeoxynucleotide probe coupled with Cy3 (GenePharma, see Table S5) was used to hybridize with the cell samples. The fluorescence intensity was observed under an A1R MP laser confocal microscope (Nikon, Tokyo, Japan) after reaction with DAPI.

**Nile red staining**

Intracellular lipid accumulation was detected by Nile Red staining (7385-67-3, Aladdin Biochemical Technology Co., Ltd., Shanghai, China). Formaldehyde-fixed AML12 cells were stained with Nile red (1 μg/mL) and imaged with a Nikon 80i.

**Immunofluorescence staining**

After permeation in PBS (0.1% Triton X-100), cells were incubated with primary antibodies (see Table S7). After DAPI staining, a Nikon 80i microscope was used for image capture.

**Histological analysis**

After fixation in 4% formaldehyde, liver samples were embedded in paraffin, and 5 μm liver sections were deparaffinized, rehydrated, and stained with hematoxylin-eosin (H&E) and Oil red O.

**Biochemical assays**

The levels of total cholesterol (TC), triglyceride (TG), alanine aminotransferase (ALT), aspartate aminotransferase (AST), low-density lipoprotein cholesterol (LDL-C), and fasting blood glucose were measured using test kits (Jiengcheng Co., Ltd., Nanjing, China).

**Western blot**

A BCA protein assay kit (Beyotime Biotechnology, Shanghai, China) was used for total protein quantification. The proteins were separated by SDS‒PAGE and incubated with appropriate primary antibodies (see Table S7). Density analysis was performed using ImageJ (National Institutes of Health, MD, USA).

**Luciferase reporter assays**

Luciferase activity was evaluated using the Multi-Luciferase HS test kit (TransGen Biotech, Beijing, China) and the Spark^TM^ multimode microplate reader (TECAN, Switzerland).

**Sucrose gradient assay**

Polysomes were extracted from AML12 cells incubated with 100 μg/mL cycloheximide (#239763, Millipore, MA, USA) and prepared in hypotonic buffer. Supercentrifugation tubes were filled with 10% to 50% (w/v) sucrose gradient solution and centrifuged at 39,000 rpm in a Beckman SW-41Ti rotor. Polymer fractions were collected from top to bottom for qRT‒PCR.

**Determination of circRNA stability**

AML12 cells were incubated with actinomycin D (5 μg/mL) for the indicated times (0 h, 12 h, 24 h, 36 h, 48 h, and 60 h). Total RNA was extracted before and after actinomycin D treatment and measured by qRT‒PCR. The half-life (t_1/2_) was calculated.

**RNA-binding protein immunoprecipitation (RIP)**

RIP was performed with the Magna RIP RNA binding protein immunoprecipitation kit (#17-700, Millipore, MA, USA) using YTHDF2 or m6A antibody. Immunoprecipitated RNA was analyzed by qRT‒PCR.

**Chromatin immunoprecipitation (ChIP)**

Ultrasonic nuclear lysates were purified and immunoprecipitated using a ChIP assay kit (#9003, Cell Signaling Technology, MA, USA). The promoter primer sequences for ChIP‒qPCR are listed in Table S6.

**DNA [agarose gel electrophoresis](javascript:;)**

A 3% agarose gel with double-stranded DNA intercalating dye (Yeasen, Shanghai, China) was prepared. DNA samples were loaded onto the gel and subjected to 75 V constant pressure electrophoresis. DNA imaging was performed using Gel-Pro Analyzer (Media Cybernetics, MD, USA).

**Histone extraction and array analysis of histone H4 modifications**

The Total Histone Extraction Kit (#EX1520, Solarbio, Beijing, China) was used to extract histones from AML12 cells. The EpiQuik™ Histone H4 Modification Multiplex Assay Kit (#P-3102, Epigentek, NY, USA) was utilized to assess modifications on histone H4. Briefly, 100 ng of histone extracts were used for site-specific identification of histone H4 modifications using a capture antibody coated in strip wells. The captured modified histones were then detected at specific sites using a detection antibody and chromogenic reagents. Absorbance measurements were performed at 450 nm using the SparkTM multimode microplate reader (TECAN). Histone H4 modification was quantified as a percentage relative to total H4.

**Statistical analysis**

The experimental results are expressed as the means±SDs. Statistical analysis was performed using GraphPad Prism software 8.0 (La Jolla, CA, USA). Two groups were compared by Student’s t test. Multiple groups were compared by one-way ANOVA. A statistically significant difference between groups is indicated by P <0.05.

**Supplementary figures**


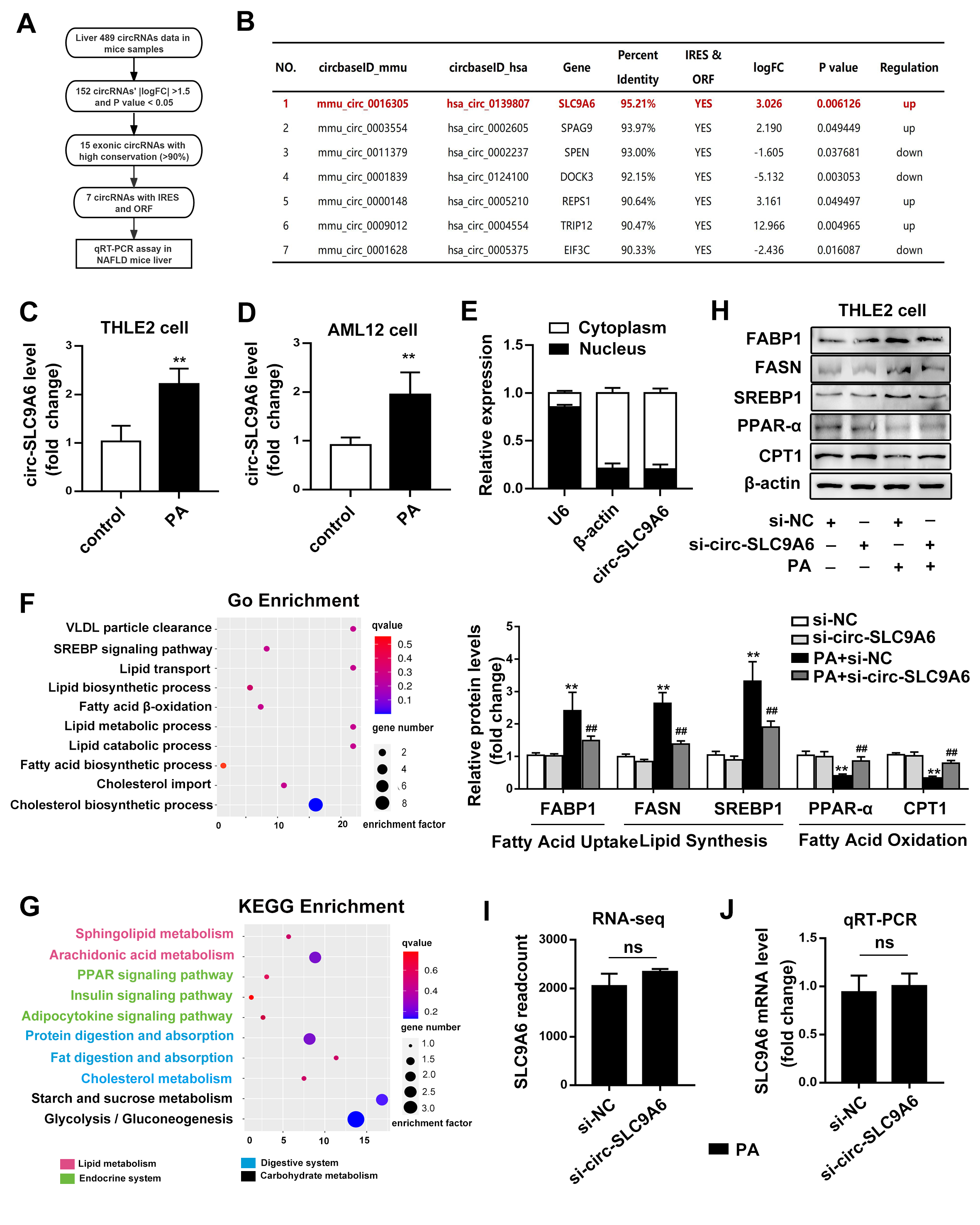


**Figure S1. Increased circ-SLC9A6 with translational function is associated with NAFLD progression in AML12 and THLE2 cells.** (A, B) Flowcharts showing the selection of highly differentially expressed circRNAs with coding potential in NAFLD. The intersection of 489 aberrantly expressed circRNAs obtained from these two datasets identified 152 candidate circRNAs (fold change > 1.5 and P < 0.05). Fifteen exonic circRNAs with high homology (>90%) were further screened. Bioinformatics analysis performed in conjunction with circRNADb, CircBank, and TransCirc online databases revealed that 7 circRNAs harbored relatively complete internal IRES and ORF sequences.(C, D) Level of circ-SLC9A6 in AML12 and THLE2 cells, n=3. (E) Cytoplasmic and nuclear RNA fractionation, n=3. (F,G) GO and KEGG pathway analysis based on RNA-seq data. (H) Protein expression of FABP1, FASN, SREBP1, PPAR-α and CPT1 in THLE2 cells, n=3. (I,J) mRNA level of SLC9A6 after circ-SLC9A6 knockdown under PA determined by RNA-seq and qRT‒PCR, n=3. ^**^p<0.01, ^##^p<0.01.


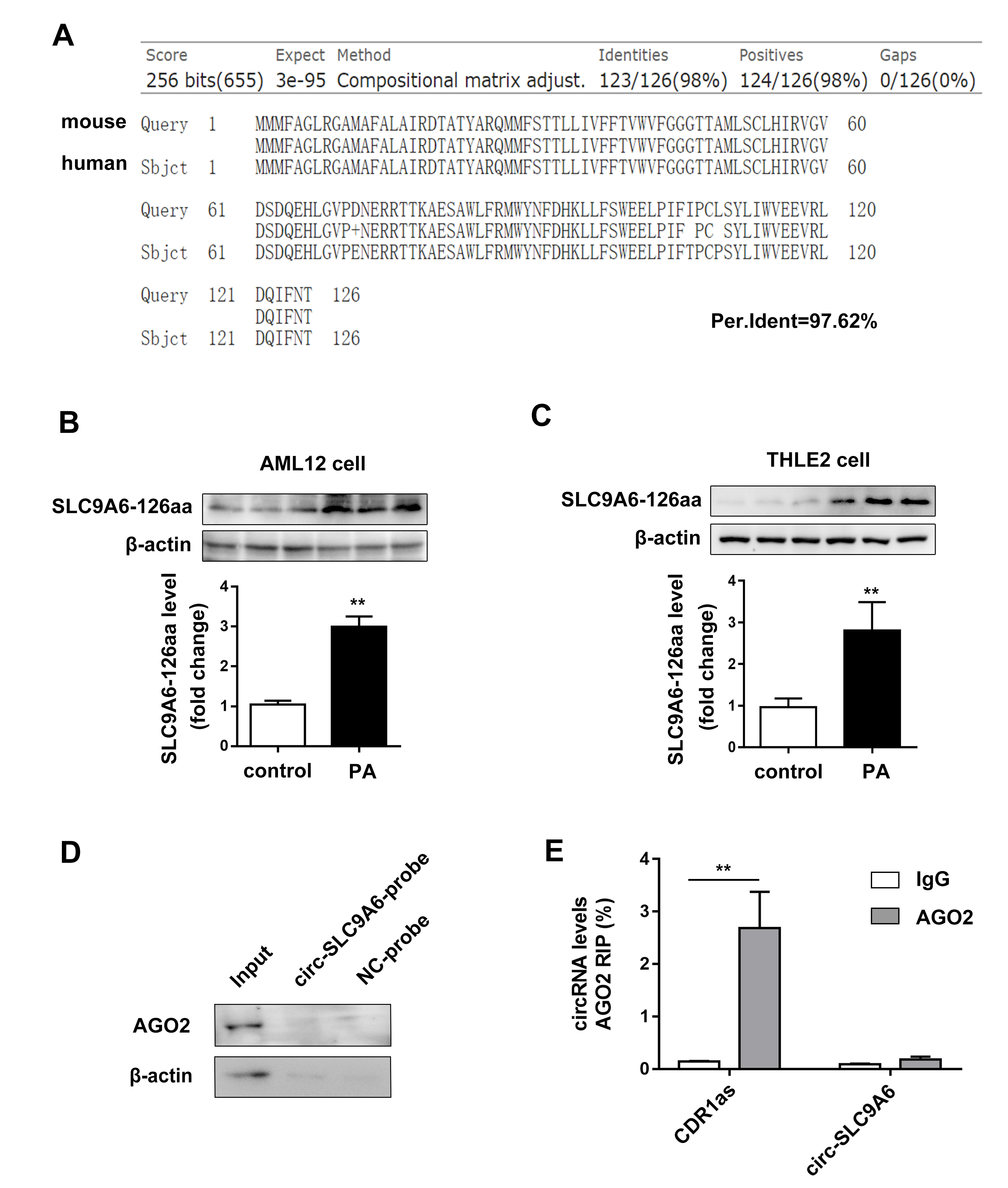


**Figure S2. Highly homologous SLC9A6-126aa is upregulated in AML12 and THLE2 cells after PA stimulation.** (A) Homology analysis of mouse and human SLC9A6-126aa based on the alignment of protein sequences from the NCBI website. (B and C) SLC9A6-126aa protein levels, n=3. (D) Pulldown assay with AML12 cells; n=3. (E) RIP assay of AML12 cells, CDR1as as a positive control; n=3.^**^p<0.01.


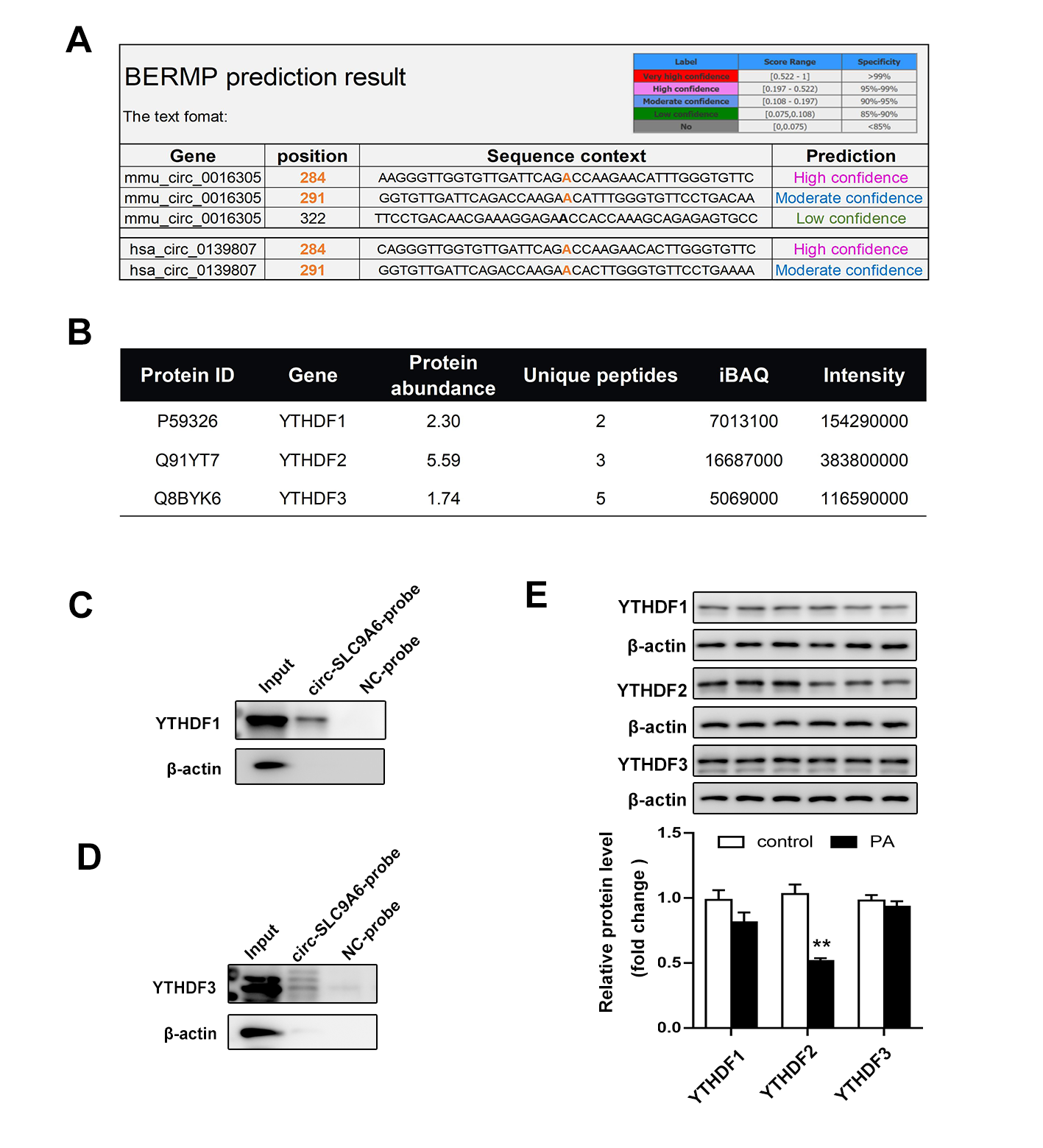


**Figure S3. Screening of YTHDFs that recognize and bind to circ-SLC9A6 in AML12 cells.** (A) The BERMP website was used to predict mouse and human circ-SLC9A6 m6A methylation sites. (B) Mass spectrophotometry showed that YTHDFs precipitated by the biotinylation probe that detects circ-SLC9A6. (C, D) YTHDF1 and YTHDF3 were pulled down and enriched with a biotinylated circ-SLC9A6 probe, n=3. (E) Expression level of YTHDF1/2/3, n=3. ^**^p<0.01.


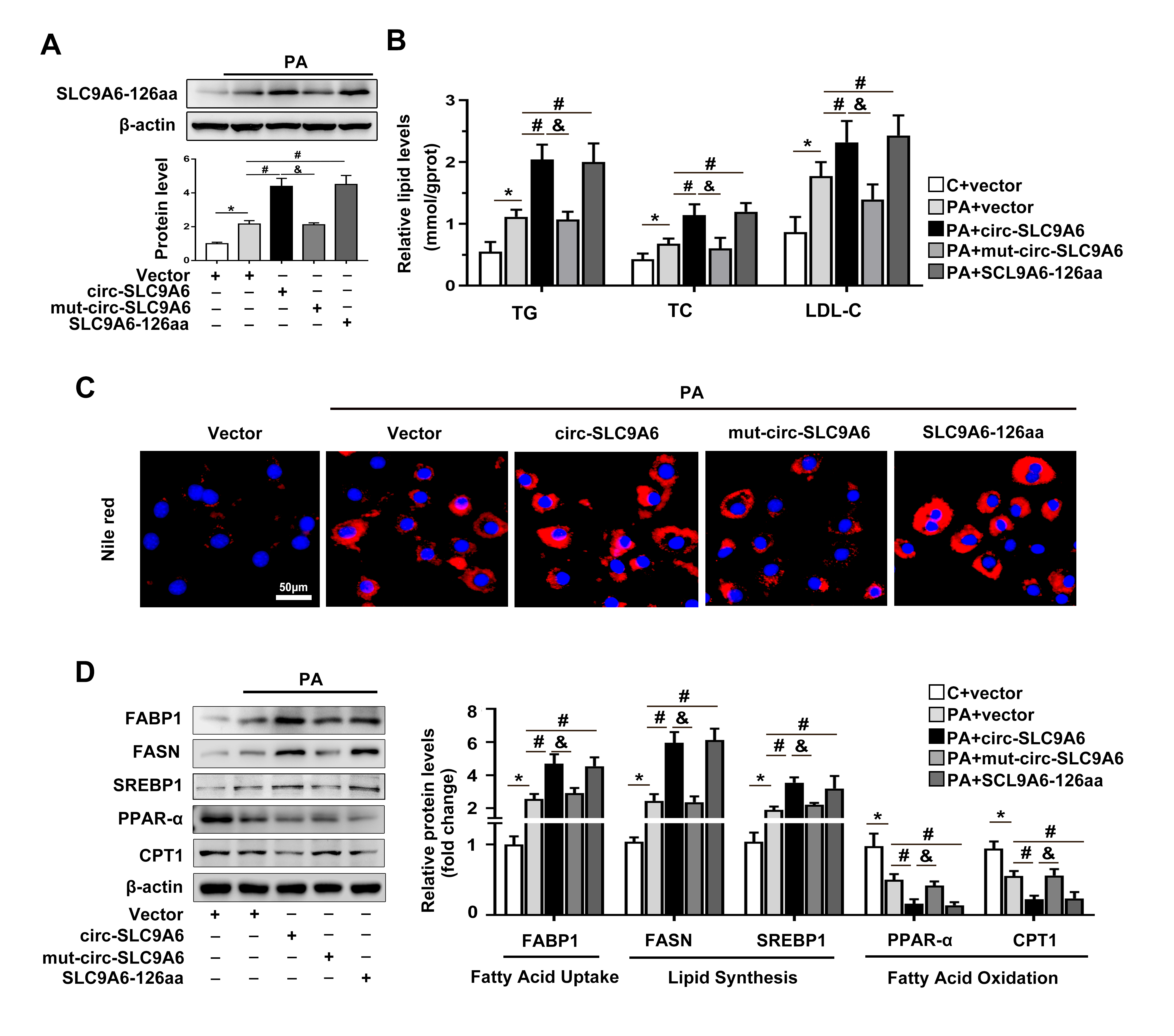


**Figure S4. SLC9A6-126aa, rather than circ-SLC9A6, aggravates lipid dyshomeostasis *in vitro*.** The circ-SLC9A6, mut-circ-SLC9A6, and SLC9A6-126aa overexpression plasmids were transfected into AML12 cells prior to PA induction. (A) SLC9A6-126aa protein level, n=3. (B)TG,TC and LDL-C level, n=6. (C)Nile red staining of AML12 cells, n=3. Scale bars=50 μm. (D) Protein expression of FABP1, FASN, SREBP1, PPAR-α, and CPT1, n=3. ^*^p<0.05, ^#^p<0.05, ^&^p<0.05.


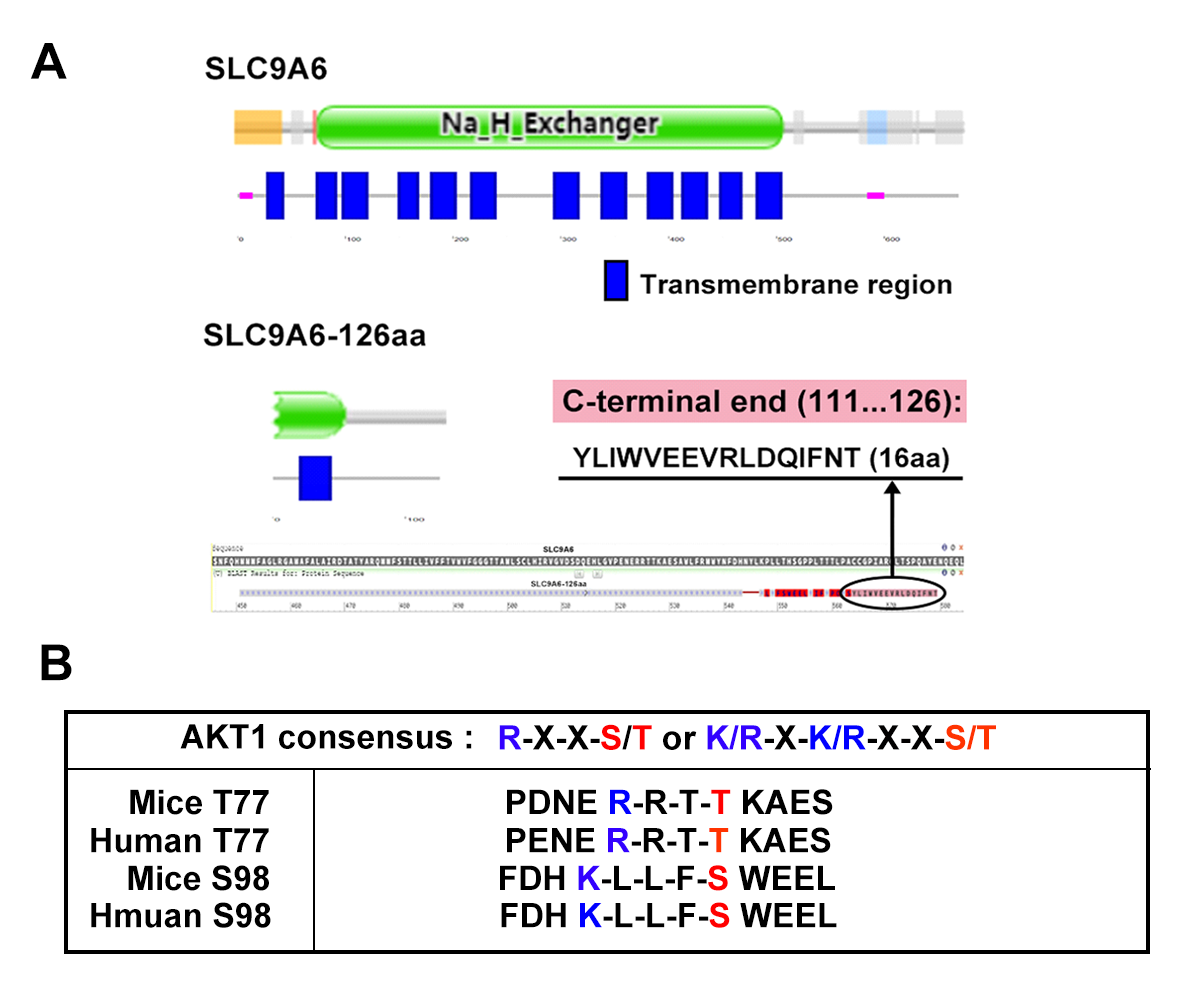


**Figure S5. Amino acid sequence analysis of SLC9A6-126aa.** (A)SMART and PFAM databases predicted that the parental gene SLC9A6 has a b_cpa1 superfamily domain containing 12 transmembrane regions. SLC9A6-126aa lacked an entire b_cpa1 superfamily domain (containing only a transmembrane region). BLAST alignment based on the amino acid sequence showed that back splicing of circ-SLC9A6 gave SLC9A6-126aa a unique 16-amino acid C-terminal structure (YLIWVEEVRLDQIFNT), which was identical in humans and mice. (B) Sequence alignment of the putative AKT1 phosphorylation site at T77 and S98 in SLC9A6-126aa from different species.


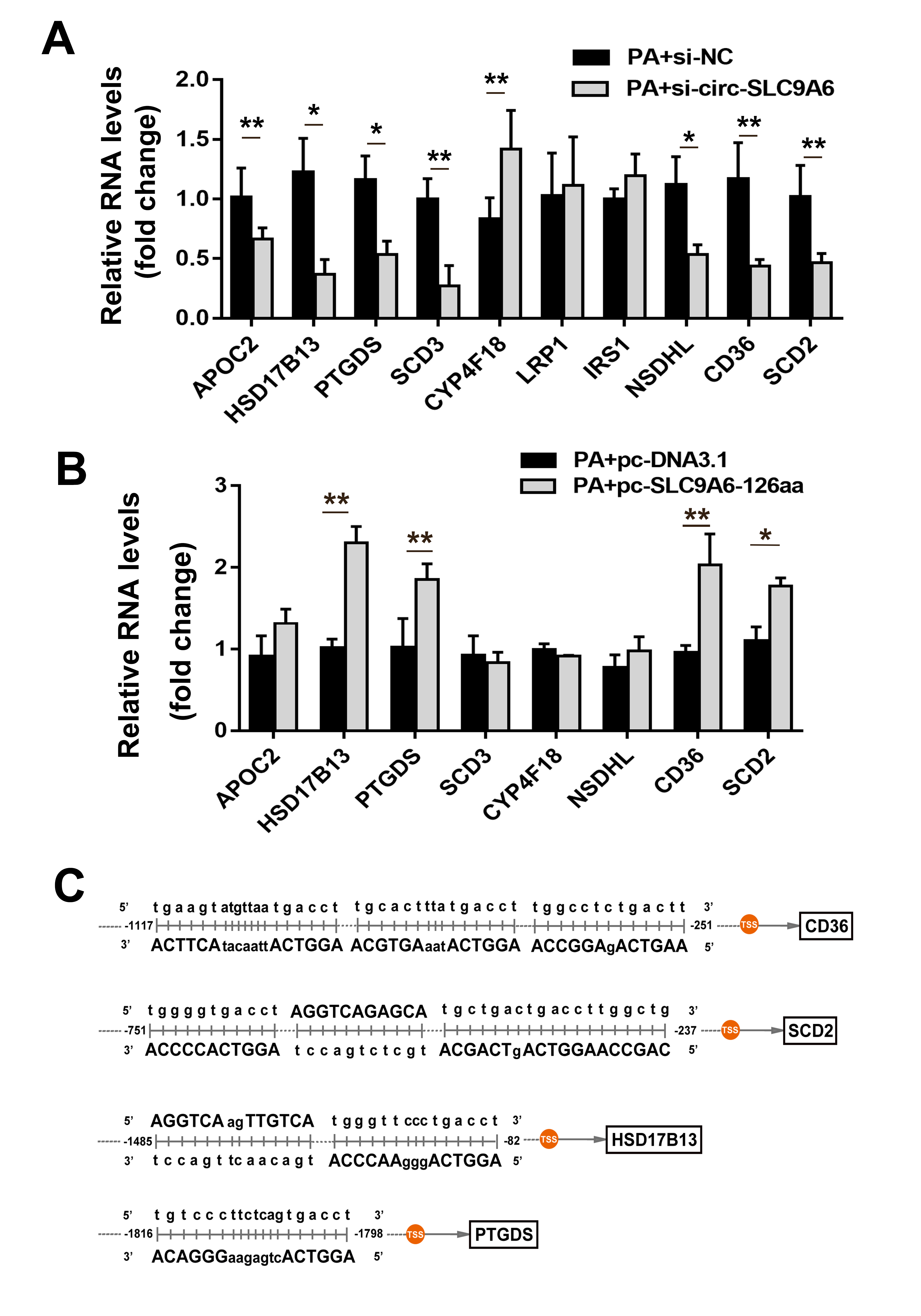


**Figure S6. Screening of target genes regulated by SLC9A6-126aa.** (A)Top 10 lipid-related RNA-seq genes expression in AML12 cells was verified by qRT‒PCR, n=3. (B)The genes regulated by SLC9A6-126aa in AML12 cells, n=3. (C) Distribution pattern of the AGGTCA-like sequence in promoter regions (-2000 bp to +100 bp from the transcription start site). ^*^p<0.05, ^**^p<0.01.


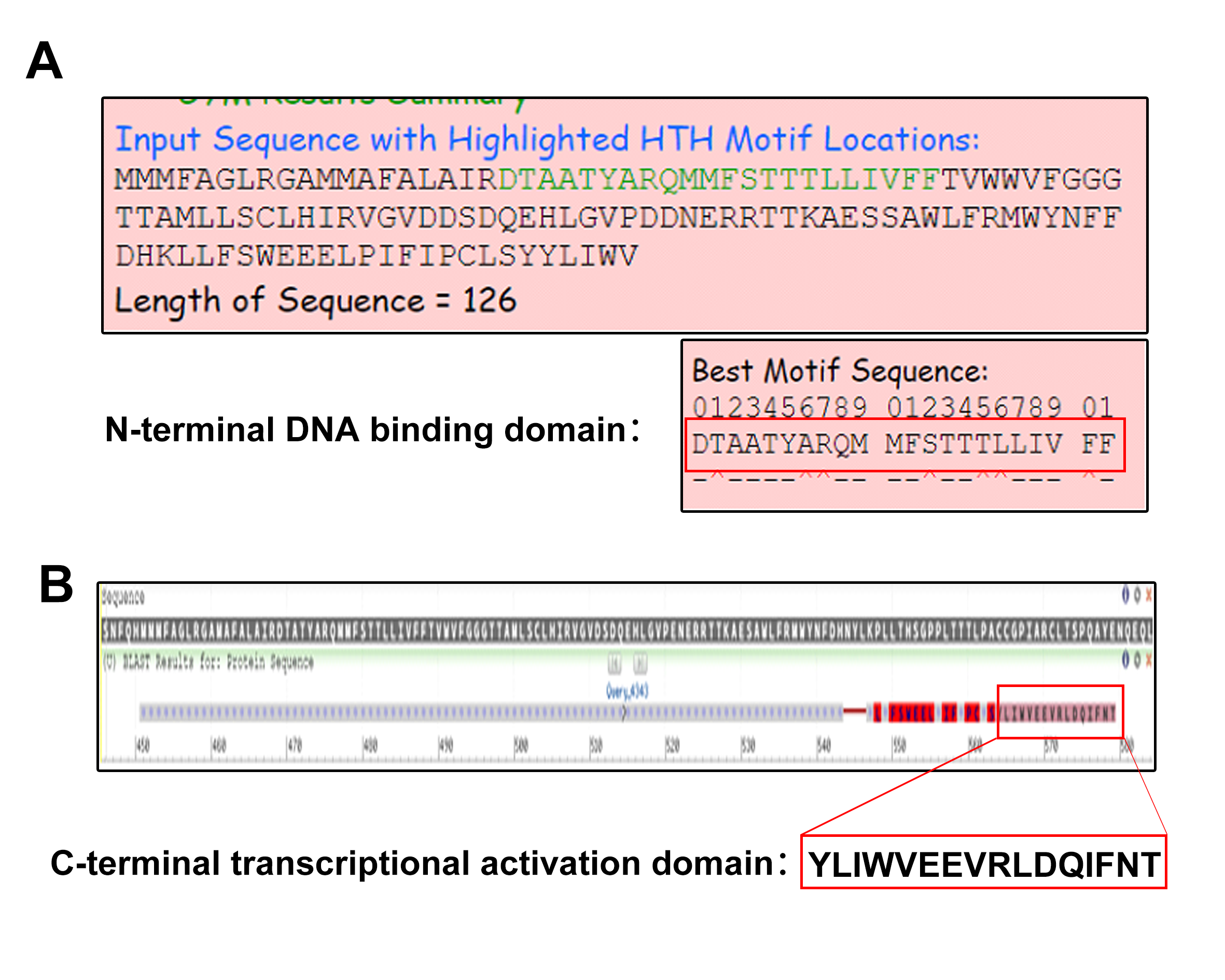


**Figure S7. Analysis of the SLC9A6-126aa functional domain.** (A) Bioinformatics analysis of a helix-turn-helix (HTH) DNA-binding motif in SLC9A6-126aa. (B) Putative C-terminal transcriptional activation domain of SLC9A6-126aa obtained by NCBI prediction.


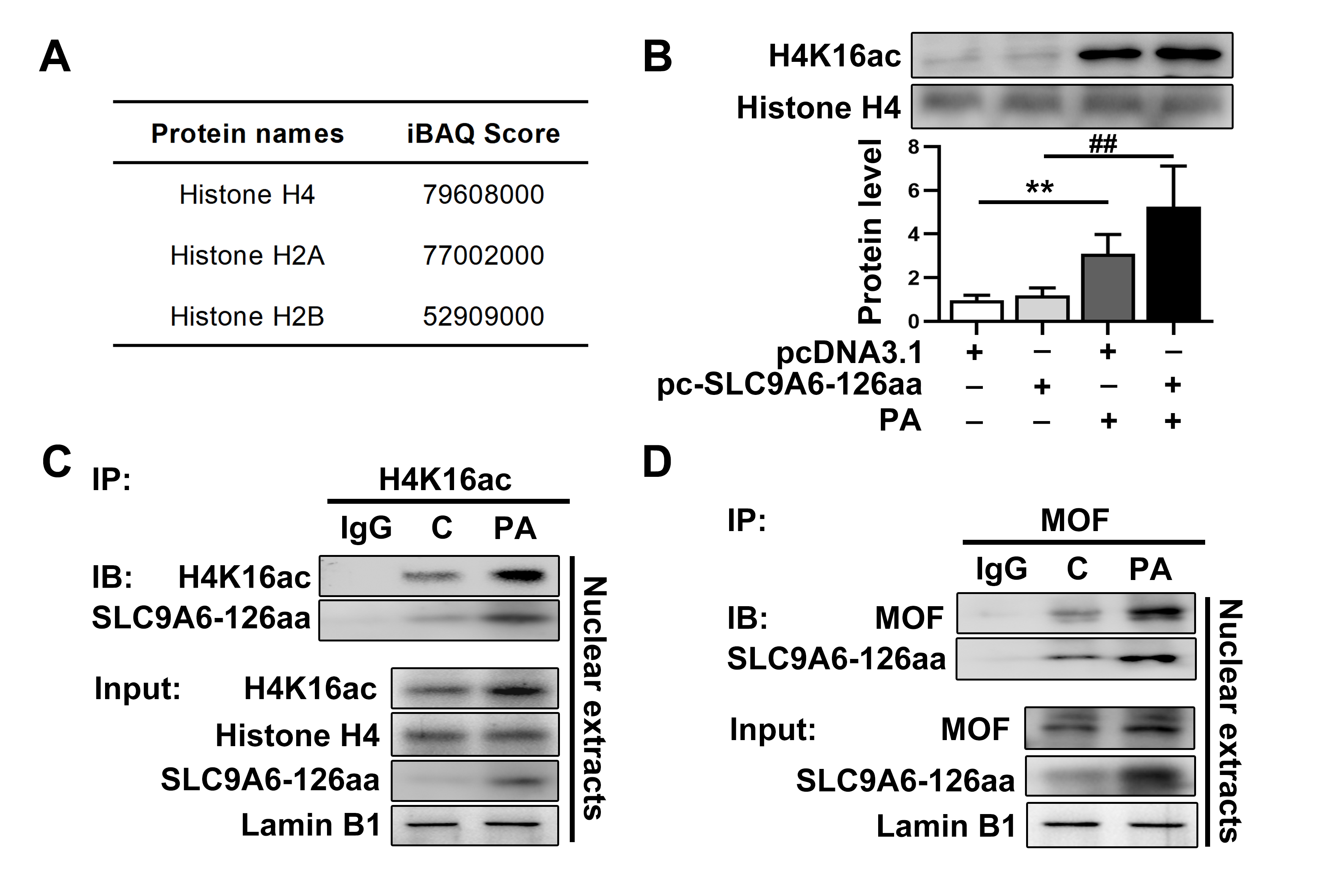


**Figure S8. SLC9A6-126aa interacts with H4K16ac and MOF in the nucleus under PA stimulation.**(A)The putative core histones binding to SLC9A6-126aa were identified through co-IP/MS analysis.(B) H4K16ac levels in AML12 cells, n=3.(C and D) Co-IP assay with AML12 cells, n=3.


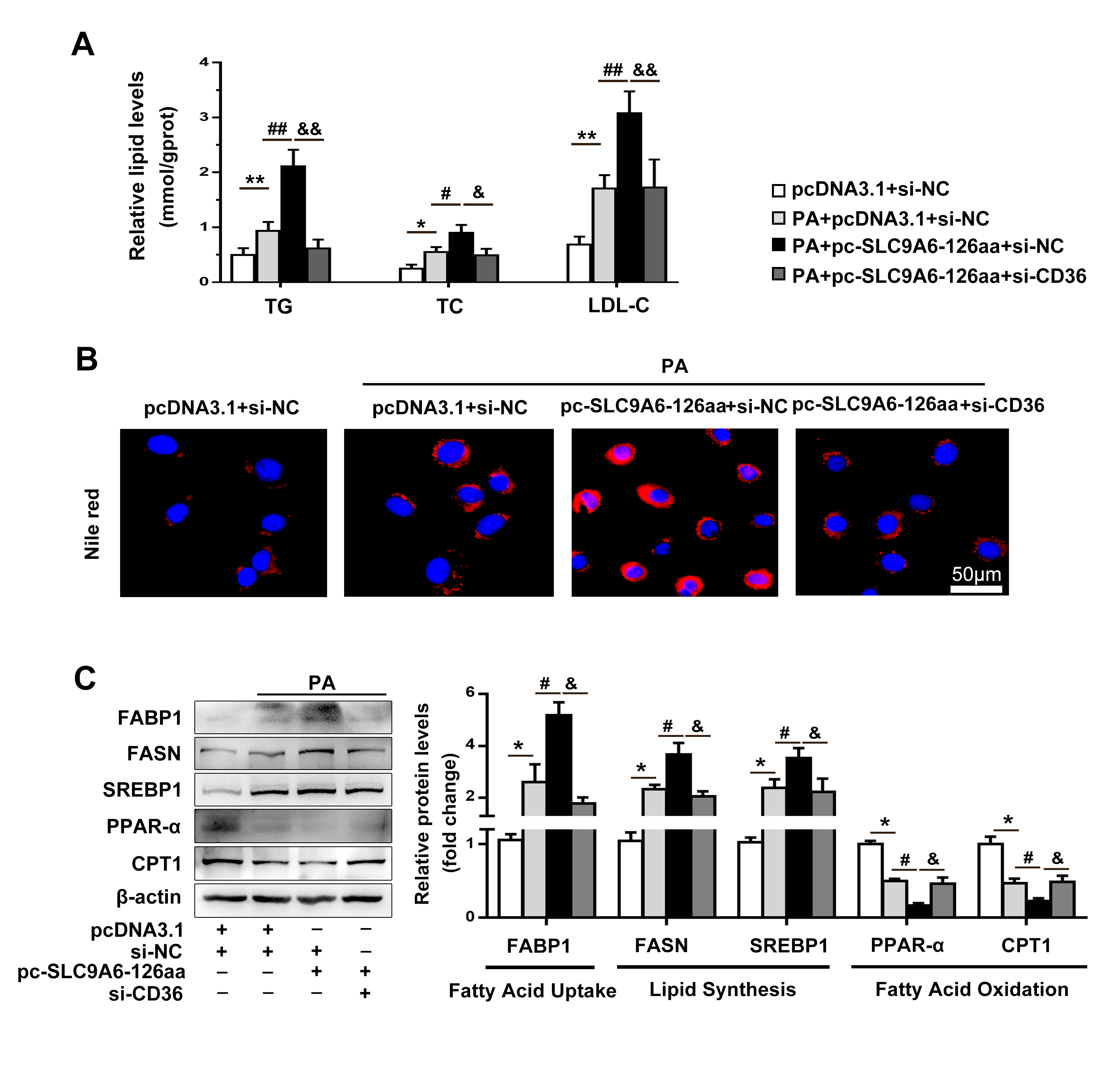


**Figure S9. CD36 deficiency reverses SLC9A6-126aa overexpression-exacerbated lipid dyshomeostasis *in vitro*.** (A-C) AML12 cells were simultaneously cotransfected with pc-DNA3.1/pc-SLC9A6-126aa plasmid and si-NC/si-CD36 and then incubated with PA. (A) TG,TC and LDL-C level, n=6. (B) Nile red staining of AML12 cells, n=3. Scale bars=50 μm.(C) Protein expression of FABP1, FASN, SREBP1, PPAR-α and CPT1, n=3. ^*^p<0.05, ^**^p<0.01, ^#^p<0.05, ^##^p<0.01, ^&^p<0.05, ^&&^p<0.01.


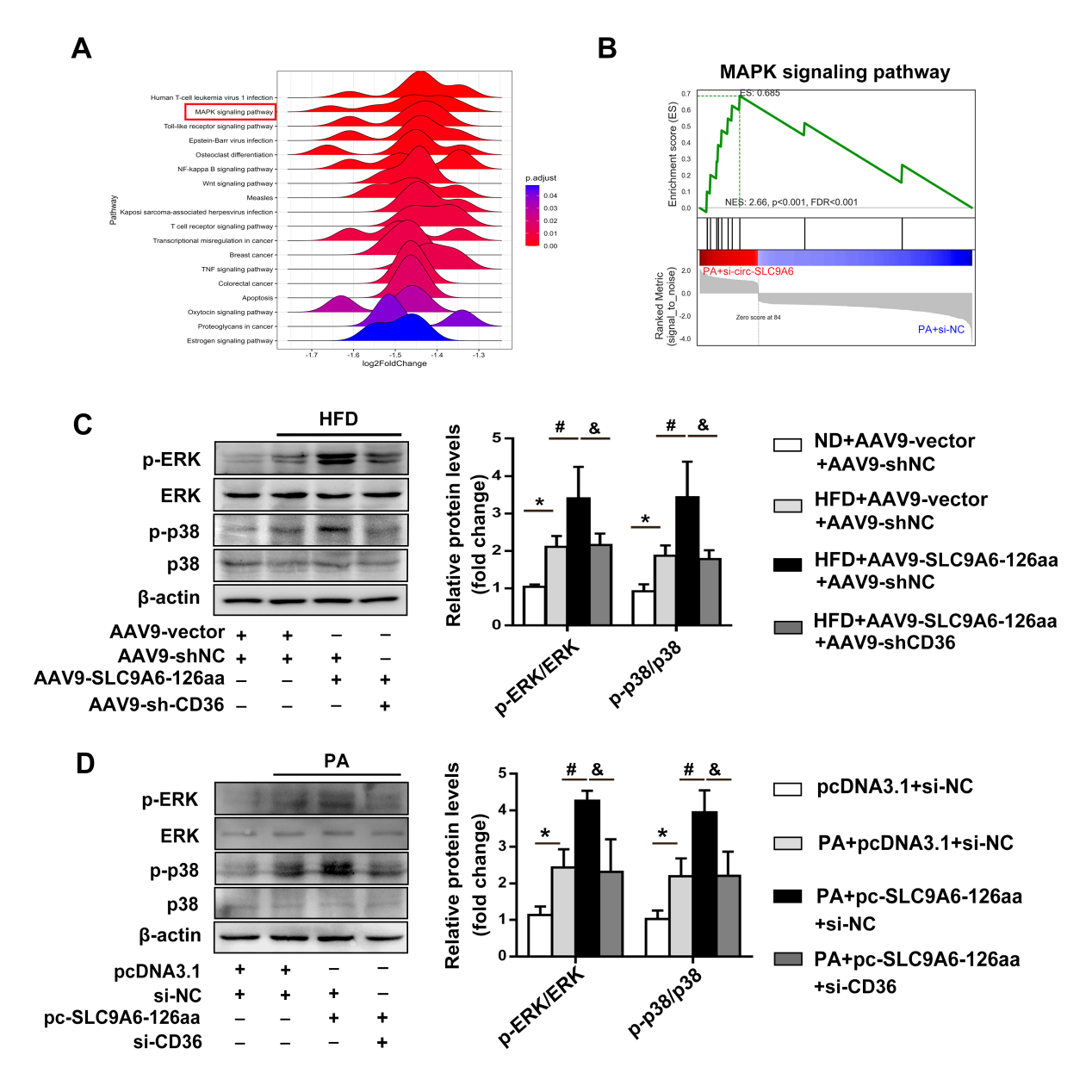


**Figure S10. SLC9A6-126aa regulates CD36/ MAPK signaling *in vitro* and *in vitro.***(A) GSEA analysis. (C and D) Protein expression of p-p38 and p-ERK, n=3. ^*^p<0.05, ^#^p<0.05, ^&^p<0.05.

**
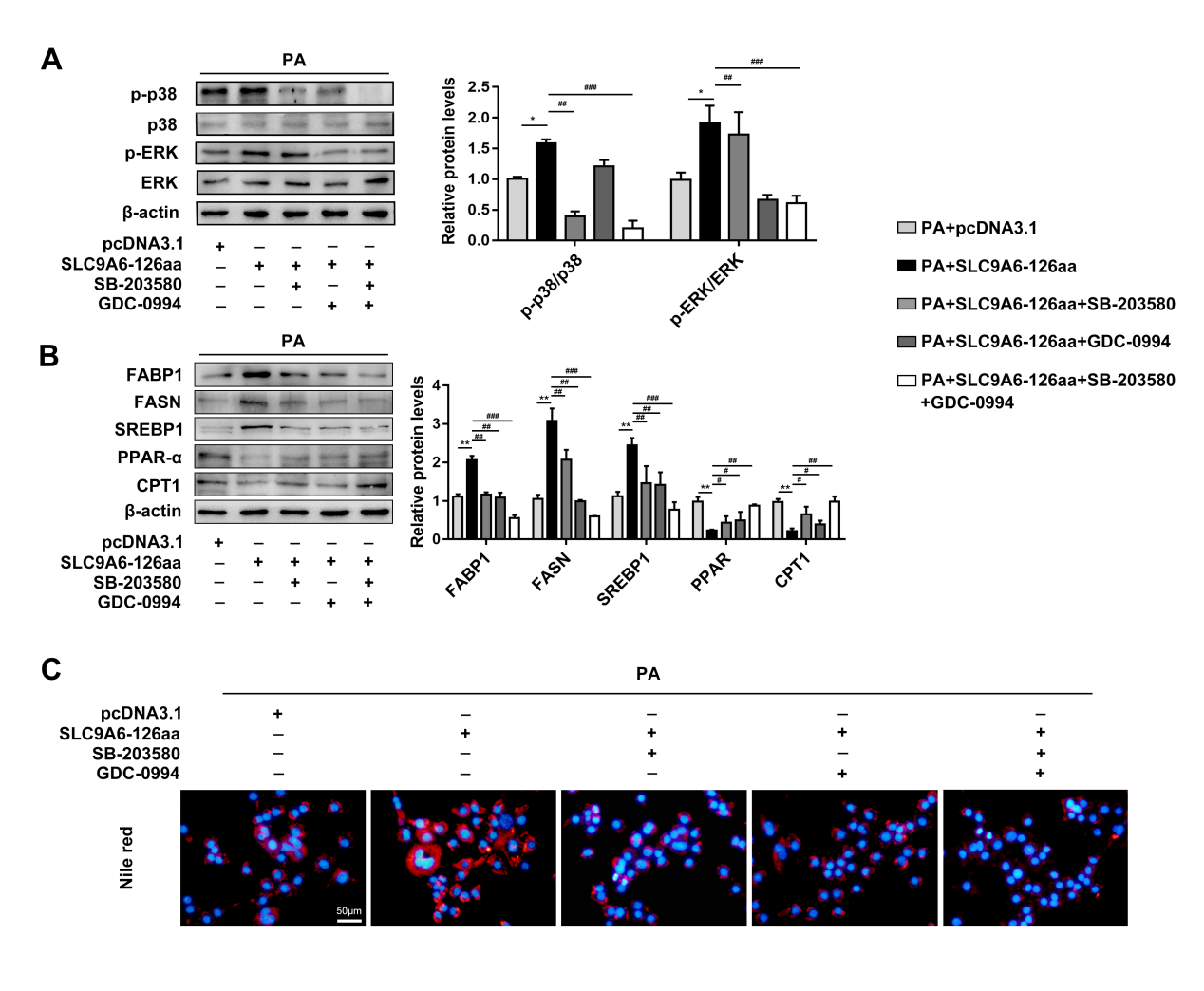
**

**Figure S11. Inhibition of the MAPK pathway alleviates SLC9A6-126aa-induced lipid dyshomeostasis *in vitro*.** AML12 cells were pretreated with SB-203580 (10μM) or GDC-0994 (1μM) for 1 h followed by PA treatment for 24 h. (A) Protein expression of p-p38 and p-ERK, n=3. (B) Protein expression of FABP1, FASN, SREBP1, PPAR-α and CPT1, n=3. (C) Nile red staining of AML12 cells, n=3. Scale bars=50 μm.^**^p<0.01, ^#^p<0.05, ^##^p<0.01,^###^P<0.0001.

# **Table S1. Potential kinases that phosphorylate SLC9A6-126aa from SCANSITE prediction**

| **Gene Info** | **Motif group** | **Colocalization** |
| --- | --- | --- |
| [AKT1](https://www.genecards.org/cgi-bin/carddisp.pl?gene=AKT1) | Basophilic serine/threonine kinase group | cytoplasm |
| AURKA | Basophilic serine/threonine kinase group | cytoplasm |
| AMPH | Src homology 3 group | cytoplasm |
| [ABL1](https://www.genecards.org/cgi-bin/carddisp.pl?gene=ABL1) | Tyrosine kinase group | cytoplasm |
| [PLK1](https://www.genecards.org/cgi-bin/carddisp.pl?gene=PLK1) | Acidophilic serine/threonine kinase group | cytoplasm |
| [PRKCE](https://www.genecards.org/cgi-bin/carddisp.pl?gene=PRKCE) | Basophilic serine/threonine kinase group | cytoplasm |
| [PRKCZ](https://www.genecards.org/cgi-bin/carddisp.pl?gene=PRKCZ) | Basophilic serine/threonine kinase group | cytoplasm |
| [YWHAZ](https://www.genecards.org/cgi-bin/carddisp.pl?gene=YWHAZ) | Phosphoserine/threonine binding group | cytoplasm |
| [PRKCD](https://www.genecards.org/cgi-bin/carddisp.pl?gene=PRKCD) | Basophilic serine/threonine kinase group | cytoplasm |

# **Table S2. Information on the top 10 lipid-related genes from RNA-seq assay**

| **Gene ID** | **Fold change** | **P value** |
| --- | --- | --- |
| APOC2 | 3.712082652 | 2.03E-16 |
| HSD17B13 | 3.309223878 | 3.44E-10 |
| PTGDS | 2.801000563 | 9.07E-11 |
| SCD3 | 2.648673225 | 2.90E-05 |
| CYP4F18 | 2.471439168 | 0.000413144 |
| LRP1 | 1.982257737 | 7.01E-51 |
| IRS1 | 1.975199761 | 4.84E-06 |
| NSDHL | 1.954132848 | 1.31E-34 |
| CD36 | 1.878402835 | 1.76E-17 |
| SCD2 | 1.786201974 | 4.32E-52 |

# **Table S3. AGGTCA-like sequence in promoters**

| **Gene ID** | **Sequence (from 5'-3')** | **Direct repeat type** |
| --- | --- | --- |
| CD36 | ..AAGTCA..AGGCCA.. | DR1 |
|  | ..AGGTCA..AGTGCA.. | DR3 |
|  | ..AGGTCA..ACTTCA.. | DR7 |
| SCD2 | ..AGGTCA..TCAGCA.. | DR1 |
|  | ..AGGTCAGAGCA.. | DR-1 |
|  | ..AGGTCACCCCA.. | DR-1 |
| HSD17B13 | ..AGGTCA..TTGTCA.. | DR2 |
|  | ..AGGTCA..AACCCA.. | DR3 |
| PTGDS | ..AGGTCA..GGGACA.. | DR7 |

# **Table S4. The demographic data of clinical samples.**

|  | **Nonsteatosis** | **Mild steatosis** | **Severe steatosis** |
| --- | --- | --- | --- |
| All | 9 | 9 | 9 |
| Male | 7 | 5 | 6 |
| Female | 2 | 4 | 3 |
| Age | 48.44±9.38 | 51.67±11.57 | 50.33±9.08 |
| Steatosis Grade (0/1/2/3) | 9/0/0/0 | 0/9/0/0 | 0/0/8/2 |
| Surgery Time | 2019.2.11 - 2022.3.18 | | |

#

# **Table S5. Sequences of siRNA and shRNA used in this study.**

| **Gene** | **sense (5'-3')** | **antisense (5'-3')** |
| --- | --- | --- |
| Mouse circ-SLC9A6 siRNA | CAACUUCGAUCAUAAAUUGTT | CAAUUUAUGAUCGAAGUUGTT |
| Human circ-SLC9A6 siRNA | CUUUGAUCAUAAGUUGCUATT | UAGCAACUUAUGAUCAAAGTT |
| Mouse CD36 siRNA | GAUCUGAAAUCGACCUUAATT | UUAAGGUCGAUUUCAGAUCTT |
| Mouse YTHDF2 siRNA | CCAUGAUUGAUGGACAGUCAGCUUU | AAAGCUGACUGUCCAUCAAUCAUGG |
| Mouse CD36 shRNA | AAGGTATATTGCTGTTGACAGTGAGCGCACATACAGAGTTCGTTATTAGTGAAGCCACAGATGTAATAACGAACTCTGTATGTGTGCCTACTGCCTCG | |
| Mouse circ-SLC9A6 probe | Cy3-CTTCCCAAGAAAATAGCAATTTATGATCGAAGTTGTACCA | |

# **Table S6. Primers used in this study.**

| **Gene** | | **Forward primer (5′–3′)** | | **Reverse primer (5′–3′)** |
| --- | --- | --- | --- | --- |
| \| **qRT-PCR primers** \| \| --- \| | | | | |
| Mouse circ-SLC9A6 | | | CTGGCTCTTTCGGATGTGGT | TGGCAGCTCTTCCCAAGAAA |
| mmu_circ_0003554 | | ACCTGCTGGAGATGGATTGC | | CTTCAGTTCTGCTTCTCTCTCTTCAA |
| mmu_circ_0011379 | | AGTGACTCCATCAGCAGCAG | | AATCCACAAAGGCAGCCACT |
| mmu_circ_0001839 | | CACATTCAGGAAGCCATGCG | | TGGGGGCAGAGTACTTGGTA |
| mmu_circ_0000148 | | ACTCCCTGAAAGCCTGATGC | | TGCACCACAAAGCTCCATGA |
| mmu_circ_0009012 | | TGCTGATCTGGGGTCACCTA | | AGCCAGTTCCGGATCTTCTT |
| mmu_circ_0001628 | | CCGACGCATGATCAGCAA | | CCAGAAGTGCATTGTGTGCAT |
| Mouse SLC9A6 | | AATGACGCGGTTGCCATAGT | | TGCAGCACCCATTGCAAA |
| Mouse CD36 | | AGATGACGTGGCAAAGAACAG | | CCTTGGCTAGATAACGAACTCTG |
| Mouse APOC2 | | ATGGGGTCTCGGTTCTTCCT | | GTCTTCTGGTACAGGTCTTGG |
| Mouse HSD17B13 | | AAAGCAGAAAAGCAGACTGGTTC | | CCCCAGTTTCCTGCATTTGT |
| Mouse PTGDS | | AGTGGTGGAGGCCAACTATG | | CCAGCCCTCTGACTGACTTC |
| Mouse SCD3 | | CTTGGATAACCACCCTGGGTG | | CTCCTCTGGAACATCACCAGCTTC |
| Mouse CYP4F18 | | CTGCATCCTCCCGTCACTG | | GGATTGTGATGTGTCCCGAAA |
| Mouse NSDHL | | TGAGCACCTCTCCCAAGATG | | ATGTTCCAGCCAATGCCAC |
| Mouse LRP1 | | ACTATGGATGCCCCTAAAA CTTG | | GCAATCTCTTTCACCGTCACA |
| Mouse IRS1 | | TGGACATCACAGCAGAATGAAGA | | GACGTGAGGTCCTGGTTGTG |
| Mouse SCD2 | | GACATTAATACCCCACTGCCA | | GCTCCCCAGTGGTGAGAACTCT |
| Mouse β-actin | | GTGACGTTGACATCCGTAAAGA | | GCCGGACTCATCGTACTCC |
| Human circ-SLC9A6 | | TGTGGTACAACTTTGATCATAAGT | | AAGTAAGAGGGACAAGGGGT |
| Human CD36 | | CTTTGGCTTAATGAGACTGGGAC | | GCAACAAACATCACCACACCA |
| Human β-actin | | CGTGGACATCCGCAAAGA | | GAAGGTGGACAGCGAGGC |
| Mouse CD36 promoter | | CGGGGTACCATACATAAAAAGCAACCCAACTC | | TCCCCCGGGCTGTGAAGAAGAAAAAGTCCTC |
| Mouse CDR1as | | TCTGCTCGTCTTCCAACATC | | AGATCAGCACACTGGAGAC |
| \| **ChIP-PCR promoter primers (Mouse)** \| \| --- \| | | | | |
| CD36/DR1 | TTGGCCATGTTCCCATCCAAG | | | TTGCTGCTACACTCCAGCATC |
| CD36/DR3 | CCCCTTCTATACTTTGTTTTCCATT | | | CTGAAAGTCTTCAGGTTCATGCTA |
| CD36/DR7 | CTTAGCCTCATGAGGTTTCCCAGG | | | GAGGGTTTACAAGATGGTGTCATCAC |
| SCD2/DR1 | GTCACCATGGTCACTGTGCACA | | | CCTAAAGGTGCCACATGTATTCGC |
| SCD2/DR-1a | ACCGCCTGCATAGGCTACA | | | GGAGGAGCTAGCTGTGCTGAA |
| SCD2/DR-1b | CGGTGCATTCACAGAGTACCTT | | | GGAAATGCCTGGCATCTCTCTTC |
| HSD17B13/DR2 | GGAGTAACTCTGGCCAAGAACAAGC | | | CACCTAAGACAGTGAGAGGCC |
| HSD17B13/DR3 | TGCTGCAGAAGAGGCAGACTTTGA | | | AACCAGGTCCAAAGGGCACATC |
| PTGDS/DR7 | CACAATGCTGCTCAGGCTGGA | | | ACGGACCTCAGAAGCTTGTCC |

# **Table S7. Primary antibodies used in this study.**

| **Antigens** | **Antibody sources** | **Species** |
| --- | --- | --- |
| SLC9A6-126aa | Merry biology,customized | Rabbit polyclonal |
| FABP1 | Proteintech,13626-1-AP | Rabbit polyclonal |
| FASN | Proteintech,10624-2-AP | Rabbit polyclonal |
| PPAR-α | ABclonal, A18252 | Rabbit polyclonal |
| CPT1 | Proteintech,15184-1-AP | Rabbit polyclonal |
| CD36 | Proteintech,18836-1-AP | Rabbit polyclonal |
| YTHDF1 | Proteintech,17479-1-AP | Rabbit polyclonal |
| YTHDF2 | Proteintech,24744-1-AP | Rabbit polyclonal |
|  |  |  |
| YTHDF3 | Proteintech,25537-1-AP | Rabbit polyclonal |
| DYKDDDDK tag | Proteintech, 66008-4-Ig | Mouse monoclonal |
|  |  |  |
|  |  |  |
| DYKDDDDK tag | Proteintech, 20543-1-AP | Rabbit polyclonal |
|  |  |  |
|  |  |  |
| AKT1 | Proteintech, 10176-2-AP | Rabbit polyclonal |
| p-AKT (S473) | Proteintech, 66444-1-Ig | Mouse monoclonal |
| Lamin B1 | Proteintech, 12987-1-AP | Rabbit polyclonal |
| β-actin | Bimake, A5092 | Mouse Recombinant Monoclonal |
| AGO2 | Abcam,ab156870 | Rabbit monoclonal |
